# Graphene Oxide-Anchored Cu–Co Catalysts for Efficient Electrochemical Nitrate Reduction

**DOI:** 10.3390/ma18112495

**Published:** 2025-05-26

**Authors:** Haosheng Lan, Yi Zhang, Le Ding, Xin Li, Zhanhong Zhao, Yansen Qu, Yingjie Xia, Xinghua Chang

**Affiliations:** 1 School of Minerals Processing and Bioengineering, Central South University, Changsha 410083, China; 2 Key Laboratory for Mineral Materials and Application of Hunan Province, School of Minerals Processing and Bioengineering, Central South University, Changsha 410083, China

**Keywords:** nitrate reduction, Cu–Co nanoalloys, ammonia synthesis

## Abstract

Electrocatalytic nitrate reduction to ammonia (ENRA) presents a promising strategy for simultaneous environmental remediation and sustainable ammonia synthesis. In this work, a Cu–Co bimetallic catalyst supported on functionalized reduced graphene oxide (RGO) was systematically designed to achieve efficient and selective ammonia production. Surface oxygen functional groups on graphene oxide (GO) were optimized through alkaline hydrothermal treatments, enhancing the anchoring capacity for metal active sites. Characterization indicated the successful formation of uniform Cu–Co bimetallic heterointerfaces comprising metallic and oxide phases, which significantly improved catalyst stability and performance. Among the studied compositions, Cu_6_Co_4_/RGO exhibited superior catalytic activity, achieving a remarkable ammonia selectivity of 99.86% and a Faradaic efficiency of 96.54% at −0.6 V (vs. RHE). Long-term electrocatalysis demonstrated excellent durability, with over 90% Faradaic efficiency maintained for ammonia production after 20 h of operation. In situ FTIR analysis revealed that introducing Co effectively promoted water dissociation, facilitating hydrogen generation (*H) and accelerating the transformation of nitrate intermediates. This work offers valuable mechanistic insights and paves the way for the design of highly efficient bimetallic electrocatalysts for nitrate reduction and ammonia electrosynthesis.

## 1. Introduction

In recent years, nitrate contamination from industrial and agricultural wastewater has exerted unprecedented pressure on ecosystems, posing significant threats to both environmental sustainability and human health [1,2]. Ammonia (NH_3_) is an essential feedstock for producing fertilizers, chemicals, and pharmaceuticals and is also a promising carbon-free energy carrier [2,3]. Currently, industrial ammonia synthesis predominantly relies on the Haber–Bosch process, which requires harsh operating conditions, with high temperatures (300–500 °C) and high pressures (200–300 atm) [4]. This method consumes substantial fossil energy and releases significant amounts of carbon dioxide, leading to severe environmental impacts [5]. With the growing global demand for ammonia, the energy consumption and environmental issues associated with the traditional Haber–Bosch synthesis process have become increasingly pressing [6]. Developing an efficient, environmentally friendly, and low-energy alternative method for ammonia production is therefore an essential requirement for sustainable societal development. Electrocatalytic nitrate reduction to ammonia (ENRA) is an attractive technology due to its minimal equipment requirements, high efficiency, and utilization of renewable electricity as the driving energy source. Moreover, ENRA selectively converts nitrate pollutants into ammonia, a valuable chemical feedstock, thus enabling waste valorization in alignment with the principles of green chemistry [7].

The selection of an appropriate catalyst is crucial for ammonia synthesis via electrocatalysis [8]. Compared to costly and scarce noble metal catalysts, transition metals such as iron, cobalt, nickel, and copper are widely employed in electrocatalytic applications [3]. Among these, copper-based materials exhibit notable activity in the nitrate-reduction reaction, making them one of the most frequently utilized catalysts. However, the relatively weak adsorption capacity of copper for nitrogen–oxygen intermediates during the ENRA process typically leads to intermediate accumulation [9,10]. Additionally, copper’s limited capacity for water dissociation often fails to satisfy the hydrogenation demands of nitrogen–oxygen species involved in the catalytic process. Therefore, introducing additional metal components is often necessary to enhance catalytic performance beyond the capabilities of a single metal catalyst [11]. For instance, Wang et al. [12] synthesized dendritic Cu_50_Ni_50_ alloys via electrodeposition and applied them to electrocatalytic ammonia synthesis. The incorporation of Ni induces electron redistribution within the alloy, resulting in an upward shift of Cu’s 3d band relative to the Fermi level, thereby enhancing the adsorption energies of reaction intermediates. Consequently, the Cu_50_Ni_50_ alloy demonstrated approximately sixfold greater capacity for nitrate (NO_3_^−^) reduction compared to a pure Cu catalyst [12]. Liu et al. [13] prepared an efficient Cu–Co@CF catalyst by modifying activated Co foam with Cu. Their findings revealed that the d-band center of the Cu–Co bimetallic material gradually shifted away from the Fermi level, significantly facilitating the hydrogenation step during the reduction process. Moreover, the reaction energy barrier for the rate-determining step decreased markedly upon Cu doping, demonstrating that introducing Cu atoms substantially promoted the reduction of NO_2_^−^ to NH_3_ [13]. These results clearly illustrate that bimetallic synergy can effectively enhance catalytic performance.

However, during prolonged reactions, metal catalysts often suffer from the aggregation of active sites and leaching of active components, leading to reduced catalytic efficiency [3,7]. Therefore, the rational design of catalysts with well-dispersed metal active centers and enhanced resistance to component leaching is crucial for efficient electrocatalytic nitrate reduction and ammonia synthesis. Graphene oxide (GO) possesses excellent electron-transport properties and abundant surface defects that can anchor metal atoms onto the carbon surface, effectively dispersing catalytic centers. Additionally, GO can suppress metal leaching during reactions, thereby enhancing the stability of the material and making it an ideal support for catalytic active sites [14,15]. Results from current research results show that various oxygen-containing functional groups (hydroxyl, epoxy, carbonyl, and carboxyl groups) can serve as anchoring sites, enabling covalent interactions with metal cations for effective loading [16,17,18]. In particular, the carbonyl (C=O) groups exhibit strong coordination affinity with Cu^2+^ and Co^2+^ ions, making them especially suitable as binding sites for metal incorporation [19,20]. Effective conversion of the functional groups on the GO surface into carbonyl (C=O) groups is crucial for achieving stable metal loading and anchoring. Previous studies have demonstrated that under strongly alkaline conditions, hydroxyl groups on the GO surface can be converted into carboxyl and carbonyl groups [21,22], However, the effect of temperature on the transformation of surface functional groups has rarely been reported. The metal-composition ratio can be precisely tuned by adjusting the metal loading, enabling a systematic investigation of the relationship between metal ratios and catalytic performance. However, studies on the catalytic behavior of GO-supported Cu–Co systems with varying metal ratios remain limited. Therefore, exploring the correlation between different Cu–Co ratios on modified GO surfaces and their catalytic performance is of significant interest.

In this study, the effect of hydrothermal temperature under alkaline conditions on the evolution of surface functional groups on GO was systematically investigated. The surface functional groups of GO were effectively tuned to optimize metal-loading conditions, leading to the synthesis of catalysts with highly dispersed active centers and good stability. In addition, the influence of different Cu/Co ratios loaded on the GO substrate on the catalyst structure and electrocatalytic performance was comprehensively studied. Using the conversion of nitrate to ammonia as a model reaction, the electrocatalytic activity and reaction mechanism of the Cu–Co/RGO catalyst were examined. This work not only deepens our understanding of the ENRA mechanism but also provides theoretical and technical support for control of nitrate pollution and resource utilization.

## 2. Catalyst Preparation

### 2.1. Chemical Reagent

Commercial graphene oxide (GO, purity 99.9%) was purchased from Shenzhen Suiheng Graphene Technology, Shenzhen, China. Cobalt (II) chloride hexahydrate (CoCl_2_·6H_2_O, purity ≥ 99.0%), Copper (II) chloride pentahydrate (CuCl_2_·5H_2_O, purity ≥ 99.0%), and sodium hydroxide (NaOH, purity ≥ 99.0%) were all obtained from Aladdin, Shanghai, China.

### 2.2. Preparation of Functional RGO Precursor

The RGO precursor was prepared using industrial-grade graphene oxide (GO) as the raw material. Specifically, 1.5 g of GO was added to a 5 mol L^−1^ NaOH solution, which was then thoroughly stirred and subjected to ultrasonic treatment at room temperature for 30 min to ensure complete dispersion in the concentrated alkaline medium. The resulting suspension was then transferred into a 50 mL polytetrafluoroethylene-lined stainless-steel autoclave and subjected to hydrothermal treatment. The heating rate was set to 10 °C/min, and the reaction was maintained for 16 h before it was allowed to naturally cool to room temperature. The extent of modification of surface functional groups on the GO was controlled by varying the hydrothermal temperature in the range 100–180 °C. The resulting precursors were designated as GO-100 °C, GO-120 °C, GO-140 °C, GO-160 °C, and GO-180 °C, with names corresponding to the specific treatment temperatures.

### 2.3. Preparation of CuxCox/RGO

The CuxCox/RGO catalysts were prepared using the previously obtained RGO precursor as the support. Specifically, 0.5 g RGO was dispersed in 20 mL of deionized water with the addition of 10 mL of ethanol to aid dispersion. Various molar ratios of CuCl_2_ and CoCl_2_ were then added as metal precursors. The resulting mixture was transferred to a 50 mL polytetrafluoroethylene-lined autoclave and subjected to hydrothermal treatment at 130 °C with a heating rate of 10 °C min^−1^ and a dwell time of 16 h. After natural cooling to room temperature, the solid products were collected by vacuum filtration, dried, and then annealed at 600 °C for 2 h under a H_2_:Ar = 10%:90% atmosphere to achieve reduction. The final catalysts were denoted as Cu/RGO, Cu_8_Co_2_/RGO, Cu_6_Co_4_/RGO, Cu_5_Co_5_/RGO, Cu_4_Co_6_/RGO, Cu_2_Co_8_/RGO, and Co/RGO, according to their respective Cu–Co loading ratios.

## 3. Experimental Section

### 3.1. Material Characterization

The phase composition and crystal structure of the materials were analyzed using X-ray diffraction (XRD) on a TD-3500 diffractometer, Dandong Tongda Science & Technology Co., Ltd., Dandong, China, equipped with a Cu-Kα radiation source (λ = 1.5406 Å). The measurements were conducted at a tube current of 200 mA and a voltage of 40 kV, with a scanning range of 5–80°. Prior to testing, the powder samples were finely ground for 20 min to ensure uniformity. Scanning electron microscopy (SEM) was employed to examine the surface morphology and microstructure of the samples using a Zeiss Sigma 300 field emission scanning electron microscope, Carl Zeiss AG, Oberkochen, Germany. The accelerating voltage was set to 30 kV, and the probe beam current was 20 nA. Prior to imaging, all samples were gold-sputtered to enhance surface conductivity. Transmission electron microscopy (TEM) was used to analyze the morphology and microstructure of the samples using a FEI Tecnai G2 F30 instrument, FEI Company, Hillsboro, OR, USA. High-resolution transmission electron microscopy (HRTEM) was employed to observe lattice fringes, FEI Tecnai G2 F30 instrument, FEI Company, OR, USA, while energy-dispersive X-ray spectroscopy (EDS) was utilized for analysis of elemental composition, Oxford Instruments, Abingdon, UK. Selected area electron diffraction (SAED) was conducted to determine the phase structure of the materials, FEI Tecnai G2 F30 instrument, FEI Company, OR, USA. All measurements were performed at an accelerating voltage of 300 kV. X-ray photoelectron spectroscopy (XPS) was employed to analyze the elemental composition and valence states of the samples using a Thermo-Scientific K-Alpha spectrometer, Thermo Fisher Scientific, Waltham, MA, USA. The binding-energy values were calibrated against the C 1s peak at 284.8 eV. Thermogravimetric analysis (TGA) was conducted to evaluate the relationship between sample mass and temperature or time. Thermogravimetric–differential scanning calorimetry (TG-DSC) measurements were performed under an air atmosphere with a gas flow rate of 10 mL/min and a heating rate of 10 °C/min. The instrument used for the test was an Netzsch STA 409PC, NETZSCH, Selb, Germany. The temperature range was set to 30 °C to 1000 °C. The phase composition and surface structure of the materials were analyzed using a confocal micro-Raman spectrometer calibrated against the characteristic peak of single-crystal silicon at 532 cm^−1^ prior to measurement. The instrument used for the test was a Renishaw In Via Qontor, Renishaw, London, UK. The metal content and elemental ratios were determined using an inductively coupled plasma atomic emission spectrometer (ICP). The instrument used for the test was a SPECTRO BLUE, SPECTRO Analytical Instruments, Kleve, Germany. For analysis, the samples were ultrasonically dissolved in 5 mol/L nitric acid, then filtered to remove solid residues. The resulting solution was then diluted with deionized water to ensure ion concentrations were within the 0–50 ppm range.

### 3.2. In Situ FTIR Test

A Nicolet is50 instrument was used for in situ infrared testing. Before the in situ test, the surface of monocrystalline silicon was gold-plated using a liquid-phase chemical method to enhance the spectral signal. The gold-plating procedure was as follows: 0.1222 g of solid sodium hydroxide was added to an aqueous solution of NaAuCl_4_ (prepared by dissolving 0.2286 g of NaAuCl_4_·2H_2_O in 3 mL of water), and the solution was subjected to an ultrasonic treatment for 1 h to prepare solution A. Separately, 0.134 g NH_4_Cl, 0.9468 g Na_2_SO_3_, and 0.6202 g Na_2_S_2_O_3_·5H_2_O were each dissolved in 50 mL of ultrapure water and subjected to ultrasonic treatment for 1 h to prepare solution B. Finally, solution A, solution B, and an additional 50 mL of ultrapure water were mixed and ultrasonicated for 2 h before use. Prior to gold plating, the monocrystalline silicon electrode was soaked in aqua regia for more than 20 min to remove surface impurities. The electrode was then polished using polishing paste, cleaned by sequential ultrasonic cleaning with acetone and deionized water, and subsequently dried. Next, the electrode was immersed in piranha solution (a mixture of 95–98% H_2_SO_4_ and 30% H_2_O_2_ at a volume ratio of 3:1) for 20 min, thoroughly rinsed with deionized water, and blow-dried. To render the surface hydrophobic, the silicon electrode was treated with 40% NH_4_F for 2–5 min, then rinsed and dried. Electroless gold plating was performed in a solution of 2% HF and gold-plating solution at a ratio of 1:4.4, maintained at 55 °C for 5–10 min. The electrode was then rinsed with deionized water and blow-dried for subsequent use. In situ infrared measurements were conducted in absorbance mode with a resolution of 16 cm^−1^ using an MCT/A detector(Xiaoxiao Photon Technology Co., Ltd, Shanghai, China), a KBr beam splitter(KBR ENERGY MANAGEMENT, Shanghai, China), a moving mirror rate of 0.4747, and an aperture setting of 200. Changes in infrared absorbance signals were monitored under varying applied voltages and electrolysis durations.

### 3.3. Electrochemical Test

All electrochemical measurements in this study, unless otherwise stated, were conducted using a single-chamber sealed electrolytic cell with a conventional three-electrode system. The electrolyte consisted of a neutral solution containing 0.1 mol L^−1^ Na_2_SO_4_ and 0.01 mol L^−1^ NaNO_3_. An Ag/AgCl electrode was used as the reference electrode, and a platinum plate served as the counter electrode. During data analysis, all measured potentials were converted to the reversible hydrogen electrode (RHE) scale. No i-R compensation was applied to the reported potentials. The specific procedures and parameters for the electrochemical test were as follows: cyclic voltammetry (CV) was performed in the potential range of −1.0–0.3 V vs. RHE with a scan rate of 10 mV s^−1^; linear sweep voltammetry (LSV) was conducted over the same potential range with a scan rate of 5 mV s^−1^; Tafel slope measurements were also carried out within −1.0–0.6 V vs. RHE at a scan rate of 1 mV s^−1^; And electrochemical impedance spectroscopy (EIS) was conducted under potentiostatic conditions to evaluate the solution resistance using a frequency range of 0.1 Hz to 100 kHz and an amplitude of 10 mV. To assess the relationship between applied potential and catalytic product distribution, constant potential electrolysis was conducted at varying voltages in 50 mL of electrolyte, with continuous stirring at 300 rpm for 2 h. After electrolysis, the samples were stored at 4 °C and analyzed within 7 days.

### 3.4. Method for Detection of Substance Concentration

The concentrations of nitrate (NO_3_^−^), nitrite (NO_2_^−^), and ammonium (NH_4_^+^) in the solution were determined using a UV-Vis spectrophotometer, based on their characteristic absorbances at specific wavelengths. These measurements were used to quantitatively evaluate the electrocatalytic performance of the materials in nitrate reduction to ammonia. The corresponding standard calibration curves are provided in the Supplementary Materials.

### 3.5. Relevant Formulas for Calculation 

During data processing, the potential was converted to the reversible hydrogen standard electrode according to Formula (1) [23]:

(1) $$E_{\mathrm{RHE}}={\;E}_{\mathrm{Ag}/\mathrm{AgCl}}+0.197+0.0592\;\times \;\mathrm{pH}\;$$

The removal ratio of nitrate was calculated according to the concentration of nitrate nitrogen before and after electrolysis, and the calculation formula is as follows:

(2) $$R\left(N-{\mathrm{NO}}_{3}^{-}\right)=\frac{C_{0}\left(N-{\mathrm{NO}}_{3}^{-}\right)-C_{t}\left(N-{\mathrm{NO}}_{3}^{-}\right)}{C_{0}\left(N-{\mathrm{NO}}_{3}^{-}\right)}$$

where R(N-NO_3_^−^) is the nitrate removal ratio, C_0_(N-NO_3_^−^) is the nitrate concentration in the initial solution, and C_t_(N-NO_3_^−^) is the nitrate concentration in the solution after electrolysis.

The selectivity of nitrite was calculated according to the product composition distribution, and the calculation formula is as follows:

(3) $$S\left(N-{\mathrm{NO}}_{2}^{-}\right)=\frac{C_{t}\left(N-{\mathrm{NO}}_{2}^{-}\right)}{C_{0}\left(N-{\mathrm{NO}}_{3}^{-}\right)-C_{t}\left(N-{\mathrm{NO}}_{3}^{-}\right)}$$

where S(N-NO_2_^−^) represents nitrite selectivity, C_0_(N-NO_3_^−^) represents nitrate concentration in the initial solution, C_t_(N-NO_3_^−^) represents nitrate concentration in the solution after electrolysis, and C_t_(N-NO_2_^−^) represents nitrite concentration in the solution after electrolysis.

The selectivity of ammonia was calculated according to the product composition distribution, and the calculation formula is as follows:

(4) $$S\left(N-{\mathrm{NH}}_{4}^{+}\right)=\frac{C_{t}\left(N-{\mathrm{NH}}_{4}^{+}\right)}{C_{0}\left(N-{\mathrm{NO}}_{3}^{-}\right)-C_{t}\left(N-{\mathrm{NO}}_{3}^{-}\right)}$$

where S(N-NH_4_^+^) is the ammonia radical selectivity, C_0_(N-NO_3_^−^) is the nitrate concentration in the initial solution, C_t_(N-NO_3_^−^) is the nitrate concentration in the solution after electrolysis, and C_t_(N-NH_4_^+^) is the ammonia radical concentration in the solution after electrolysis.

Assuming that two electrons are consumed to produce an NO_2_ molecule, FE (N-NO_2_^−^) can be calculated as follows [24]:

(5) $$\mathrm{FE}\left(N-{\mathrm{NO}}_{2}^{-}\right)=\frac{n\times F\times V\times C_{t}\left(N-{\mathrm{NO}}_{2}^{-}\right)}{M\times Q\times 1000}$$

where FE (N-NO_2_^−^) is the Faraday efficiency of ammonia production, n is the number of electrons in the reaction, F is the Faraday constant (96,485 C/mol), V is the volume of the solution, M is the molecular mass of nitrogen (14 g/mol), and C_t_(N-NO_2_^−^) is the concentration of nitrite in the solution after electrolysis.

Assuming that eight electrons are consumed to produce an NH_4_^+^ molecule, FE(N-NH_4_^+^) can be calculated as follows [25]:

(6) $$\mathrm{FE}\left(N-{\mathrm{NH}}_{4}^{+}\right)=\frac{n\times F\times V\times C_{t}\left(N-{\mathrm{NH}}_{4}^{+}\right)}{M\times Q\times 1000}$$

where FE(N-NH_4_^+^) represents the Faraday efficiency of ammonia production, n represents the number of electrons in the reaction, F is the Faraday constant (96,485 C/mol), V represents the volume of the solution, M represents the molecular mass of nitrogen (14 g/mol), and C_t_(N-NH_4_^+^) represents the concentration of nitrite in the solution after electrolysis.

Based on the results obtained from UV–Vis spectrophotometry, the ammonia yield can be calculated using the following equation:

(7) $$Y\left(N-{\mathrm{NH}}_{4}^{+}\right)=\frac{V\times C_{t}\left(N-{\mathrm{NH}}_{4}^{+}\right)}{M}$$

where V represents the volume of the solution, Ct(N-NH_4_^+^) represents the concentration of nitrite in the solution after electrolysis, and M represents the mass of the catalyst.

The Turnover Number (TON) was used to evaluate the utilization efficiency of the catalytic active sites and was calculated using the following equation:

(8) $$\mathrm{TON}=\frac{N_{\mathrm{product}}}{N_{\mathrm{active}\;\mathrm{site}}}$$

where N_product_ represents the number of moles of the target product NH_4_^+^ and N _active site_ represents the number of moles of catalytic active sites, calculated based on the moles of the metal components.

## 4. Results and Discussion

### 4.1. Regulation of Surface Oxygen-Containing Functional Groups on GO

The structural changes in graphene oxide (GO) during hydrothermal treatment were analyzed by X-ray diffraction (XRD). As shown in Figure S1, untreated GO exhibited a distinct diffraction peak at 2θ = 12.28°, corresponding to the (001) plane of GO [15]. Following hydrothermal treatment, this peak disappeared and a broad, low-intensity peak characteristic of amorphous carbon emerged around 23°. This indicates that the oxygen-containing functional groups on the GO surface undergo significant transformation during hydrothermal processing, leading to a reduction in the interlayer spacing of the graphene sheets [21].

**Figure 1:**
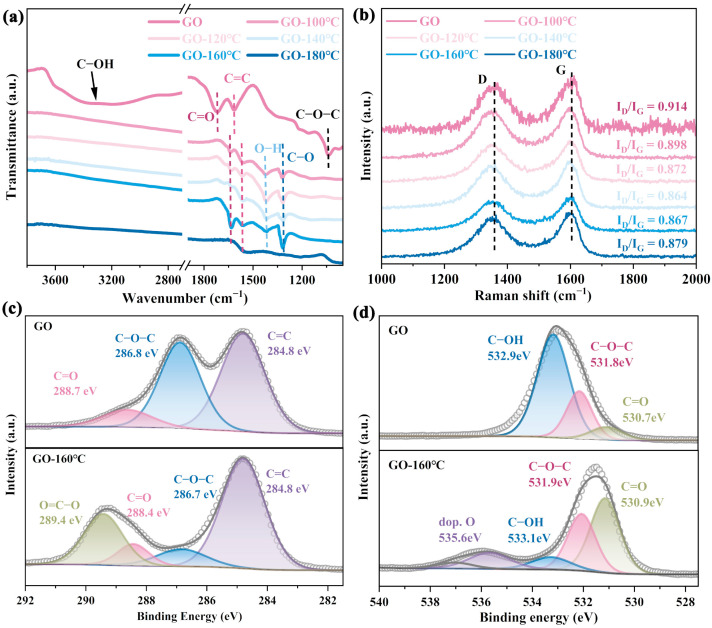
(**a**) FTIR spectra of GO after hydrothermal treatment at different temperatures. (**b**) Raman spectra. (**c**) C 1s fitting analysis before and after hydrothermal treatment. (**d**) O 1s fitting analysis before and after hydrothermal treatment. The gray circle represents the original test data, and the gray line represents the fitting analysis data.

FTIR was performed to investigate the changes in oxygen-containing functional groups on the GO surface after hydrothermal treatment, As shown in Figure 1a, the pristine graphene oxide exhibited a broad absorption band in the range 3200–3600 cm^−1^, which can be attributed to the combined contributions of water molecules, surface hydroxyl groups, and the O–H stretching vibrations of carboxylic acid groups [26]. The absorption peak at 1725 cm^−1^ corresponds to the C=O stretching vibration, while the peak at 1610 cm^−1^ can be attributed to the C=C stretching vibration within the graphene framework [27]. Additionally, the peak at 1052 cm^−1^ is associated with the C–O stretching vibration of surface epoxy groups [28]. After hydrothermal treatment under concentrated alkaline conditions, the intensity of the O–H stretching vibration band in the range of 3200–3600 cm^−1^ decreased significantly, indicating the removal of a large number of unstable surface hydroxyl groups. Similarly, the intensity of the C–O stretching vibration peak at 1052 cm^−1^ was also reduced. This can be attributed to the ring-opening of epoxy groups, which are readily transformed into new oxygen-containing functional groups. As a result, the original epoxy/alkoxy structures were disrupted and converted into different oxygen-containing functional groups during the alkaline hydrothermal process. Correspondingly, a new absorption peak emerged at 1318 cm^−1^; this can be attributed to oxygen-containing functional groups formed through the ring-opening of epoxy groups [21]. Additionally, a characteristic peak appeared at 1653 cm^−1^, with its intensity gradually increasing as the hydrothermal temperature rose. This peak corresponded to the C=C skeletal vibrations of aromatic rings, as well as to structures associated with carboxyl (–COO^−^) or carbonyl (C=O) groups. Notably, this peak exhibited a red shift compared to that observed prior to hydrothermal treatment, which may be attributed to the formation of conjugated structures or carboxylate configurations involving partial carbonyl groups under alkaline conditions [21]. The characteristic peak at 1558 cm^−1^ corresponds to the asymmetric stretching vibration of the carboxylate group (–COO^−^). Under alkaline conditions, carboxylic acid groups are readily deprotonated to form carboxylate structures, giving rise to this distinct asymmetric stretching peak. The absorption peak at 1415 cm^−1^ is typically associated with the symmetric stretching vibration of carboxylate groups (–COO^−^) or the bending vibration of hydroxyl groups (O–H). When the hydrothermal temperature reaches 180 °C, the characteristic peaks in the FTIR spectrum are significantly weakened or even disappear. This phenomenon may be attributed to two possible factors: (1) the elevated hydrothermal temperature alters the structure of graphene oxide, leading to the attenuation or loss of functional-group signals in the FTIR spectrum; (2) under high-temperature alkaline conditions, localized reactive centers may form, inducing secondary oxidation or the introduction of other oxygen-containing functional groups (e.g., lactones), which may transform conventional groups such as –C=O and result in changes in the FTIR features [29,30]. As a result, conventional functional groups (e.g., –OH, C=O) may be transformed into new oxygen-containing structures, causing the weakening or disappearance of their corresponding signals without a significant decrease in the overall oxygen content (Table S1).

The chemical and structural properties of the RGO precursors were further analyzed by Raman spectroscopy. As shown in Figure 1b, the D band and G band of carbon appeared at 1350 cm^−1^ and 1600 cm^−1^, respectively [26,31]. With increasing hydrothermal temperature, the intensity ratio of the D to G band (R = I_D_/I_G_) initially decreased and then increased. When the hydrothermal temperature was in the range 100–140 °C, the R value decreased from 0.914 to 0.864 (Figure S3). Simultaneously, the C–OH peak in the FTIR spectrum weakened significantly and the XPS analysis showed a gradual decrease in the contents of C–OH and C–O–C species (Figures S3 and S4), indicating a reduction in surface defects due to the removal of unstable hydroxyl groups and a decrease in epoxy functional groups. However, as the temperature increased further to 140–180 °C, the R value rose from 0.864 to 0.879 (Figure S3). During this stage, XPS results reveal enhanced signals of –O–C=O and –C=O groups, suggesting that new defect sites were introduced via the formation of carboxyl and carbonyl functional groups on the graphene surface, thereby increasing the overall defect density. Notably, at 180 °C, the intensity of –O–C=O and –C=O peaks decreased, while signals corresponding to other oxygen-containing groups (Dop. O) were enhanced (Figure S4), a finding consistent with the FTIR and Raman spectroscopy results. The morphological changes in GO before and after hydrothermal treatment were examined by scanning electron microscopy (SEM). As shown in Figure S2, the surface of the material exhibited a significant increase in the number of folds and pits following hydrothermal treatment. This can be attributed to gas evolution or localized alkaline corrosion under high-temperature hydrothermal conditions (180 °C). Additionally, the alkaline environment may locally etch the graphene sheets, resulting in further pore formation and pronounced surface folding. Such a wrinkled surface morphology is beneficial for the subsequent loading and anchoring of metal species. It is also noteworthy that the R of GO after hydrothermal treatment was lower than the original value of 0.92, indicating a reduction in the number of structural defects due to the removal of most surface hydroxyl and epoxy groups.

The distribution and chemical states of carbon and oxygen elements on the material surface were further analyzed using X-ray photoelectron spectroscopy (XPS). Based on the XPS data, the surface C:O ratio was calculated, as summarized in Table S1. As the temperature of hydrothermal treatment increased, the C:O ratio progressively decreased from 2.03 to 0.98. This trend indicates that low-oxygen-content functional groups were gradually converted into higher-oxygen-content groups, such as carbonyl and carboxyl groups, as the treatment temperature increased. As shown in Figure S4 and Figure 1c, the characteristic peaks at approximately 284.8 eV, 286.5 eV, 288.5 eV, and 289.5 eV correspond to C=C bonds, C–O single bonds from surface hydroxyl and epoxy groups, C=O double bonds in carbonyl groups, and O–C=O bonds in carboxyl groups, respectively [32,33]. The relative proportions of these functional groups are summarized in Table S2. As the hydrothermal temperature increased to 160 °C, the proportion of low-oxygen-content groups such as hydroxyl and epoxy (C–O) groups decreased to 24.34%, while the proportion of high-oxygen-content groups such as carbonyl and carboxyl groups increased to 56.15%. When the hydrothermal temperature increased to 180 °C, the proportion of C–O single bonds on the material surface further decreased, as did carboxyl group content. However, the overall surface C/O ratio dropped to 0.98, suggesting that excessive treatment temperature results in further transformation of oxygen-containing functional groups. This may be attributed to secondary oxidation processes that led to the formation of additional oxygen-containing functional groups that were not originally present.

For O 1s high-resolution XPS fitting analysis, as shown in Figure 1d and Figure S5, the characteristic peaks at 530.7 eV, 531.8 eV, 533.1 eV, and 535.2 eV correspond, respectively, to the following: C=O double bonds in carbonyl and carboxyl groups; C–O–C single bonds linked to aliphatic carbon; C–OH single bonds associated with aromatic carbon; and doped oxygen or other oxygen-containing groups (denoted as Dop.O) within or adjacent to the graphene plane [14,21,33]. These results, consistent with the FTIR and C 1 s fitting analyses, demonstrate that increasing the hydrothermal temperature promoted the enrichment of high-oxygen-content functional groups on the GO surface. However, at 180 °C, the content of carboxyl groups began to decline, while the increase in doped oxygen species suggests the possible formation of peroxides. Overall, the analysis indicates that hydrothermal treatment at 160 °C yields the optimal distribution of targeted functional groups, making it a suitable condition for preparing precursors for metal loading.

### 4.2. Preparation and Characterization of CuxCox/RGO

**Figure 2:**
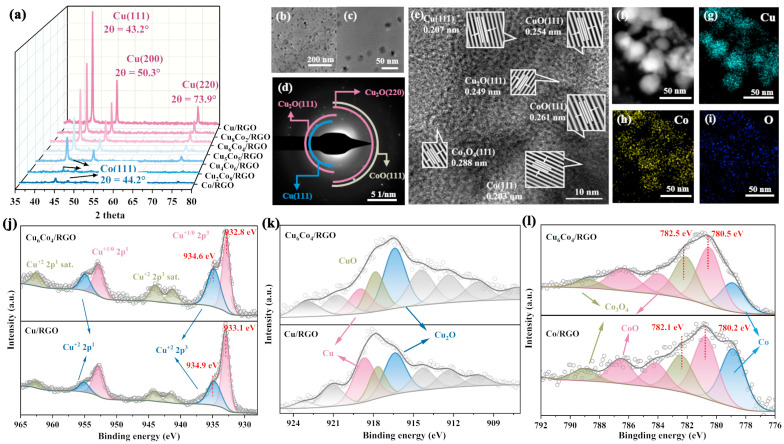
Material characterization analysis: (**a**) XRD patterns; (**b**,**c**) TEM images showing morphology; (**d**) SAED pattern analysis; (**e**) HRTEM image showing surface lattice fringes; (**f**–**i**) EDS elemental-distribution maps; (**j**) Cu 2p high-resolution XPS-fitting results; (**k**) Cu LMM Auger-fitting analysis; (**l**) Co 2p high-resolution XPS-fitting results. The gray circle represents the original test data, and the gray line represents the fitting analysis data.

Using the RGO precursor obtained from the aforementioned experiment as the support, a series of samples with varying Cu–Co loading ratios were prepared. The samples were named based on their Cu/Co molar ratios, which were quantitatively determined by inductively coupled plasma (ICP) analysis (Table S3). The phase compositions of the samples were characterized by XRD. As shown in Figure 2a, three prominent diffraction peaks corresponding to Cu (111), (200), and (220) planes were observed at 43.2°, 50.3°, and 73.9°, respectively [34,35]. With a progressive decrease in the Cu content and a corresponding increase in Co content, the intensity of the Cu-related peaks diminished, while the characteristic peak of Co (111) emerged at 44.2° [36]. The experimental results confirmed the successful loading of both Cu and Co metals, and the desired Cu–Co ratios were effectively achieved by adjusting the respective precursor addition ratios.

FTIR was used to analyze the changes in the surface functional groups of materials with different Cu–Co ratios. As shown in Figure S6a, after metal loading, the characteristic peaks associated with oxygen-containing functional groups at 1318 cm^−1^, 1653 cm^−1^, and 1558 cm^−1^, corresponding to carboxylate (–COO^−^) and carbonyl (C=O) structures, disappeared, while only a weak absorption peak remained at 1415 cm^−1^. This peak is typically associated with the symmetric stretching vibration of carboxylate groups (–COO^−^) or the bending vibration of hydroxyl groups (O–H). These results indicate that Cu^2+^ and Co^2+^ in the solution effectively interacted with surface oxygen-containing groups during the metal-loading process. These metal ions interacted with oxygen-containing functional groups on the RGO surface and bound to the surface through electrostatic adsorption or chemical coordination. Subsequently, metal loading and anchoring were achieved via high-temperature annealing and reduction. The chemical structural characteristics of the resulting materials were further analyzed using Raman spectroscopy (Figure S6b,c). Characteristic Raman peaks corresponding to Cu_2_O, CoO, and other metal oxides were observed at 385 cm^−1^, 440 cm^−1^, and 680 cm^−1^, respectively, confirming the successful loading of metal species [37,38,39]. The surface morphology of the materials was examined by scanning electron microscopy (SEM), as shown in Figure S7. Pristine RGO exhibited numerous folds and pits, attributed to the high density of surface defects. After metal loading, distinct metal nanoparticles became visible on the material surface. With increasing Co content, the extent of metal loading increased and reached a maximum at Cu_6_Co_4_/RGO. However, upon further increases to the Co proportion, both the quantity and size of metal particles on the surface decreased. This phenomenon may be attributed to the elevated concentration of Co^2+^ ions, which enhanced the interaction between the metal ions and oxygen-containing functional groups on the RGO surface. Such interactions could accelerate metal nucleation and growth, potentially resulting in agglomeration and detachment of metal particles. Energy-dispersive X-ray spectroscopy (EDS) was employed to analyze the elemental composition and spatial distribution of metal particles on the surface of Cu_6_Co_4_/RGO. As shown in Figure S8, both Cu and Co elements were uniformly distributed across the material surface, along with a significant presence of oxygen. This indicates that the metals were primarily present in the form of metal oxides anchored on the RGO surface.

High-resolution transmission electron microscopy (HRTEM) was used to further investigate the crystal structure of the metal particles on the surface, while EDS was employed to analyze the elemental distribution. The results are shown in Figure 2b–i. The material surface was uniformly decorated with metal nanoparticles ranging from 20 to 40 nm in diameter and composed of Cu, Co, and O elements. These nanoscale particles were evenly dispersed, offering abundant active sites for catalytic reactions. Lattice-fringe analysis revealed the presence of various metal oxide phases at the edges of the particles, including CuO (111) with a spacing of 0.254 nm, Cu_2_O (111) at 0.249 nm, Co_3_O_4_ (111) at 0.288 nm, and CoO (111) at 0.261 nm [40]. In contrast, the particle interiors contained metallic single crystals, such as Cu (111) at 0.207 nm and Co (111) at 0.203 nm [41]. This can be attributed to the higher susceptibility of surface metals to oxidation compared to those in the core. Additionally, spatially staggered distributions of these metal oxides, as shown in Figure S9, result in the formation of a heterogeneous metal-oxide interface. This unique heterostructure enhances the adsorption and transformation of nitrogen- and oxygen-containing intermediates, thereby promoting the ENRA process.

XPS was further employed to analyze the elemental composition and valence states on the surface of Cu_6_Co_4_/RGO, with Cu/RGO and Co/RGO used as reference samples. As shown in the full survey spectra in Figure S10a, characteristic peaks corresponding to Cu, Co, and O were clearly observed on the surface of Cu_6_Co_4_/RGO. In contrast, only the peaks corresponding to individual metal elements were present in Cu/RGO and Co/RGO, confirming the successful co-deposition of Cu and Co on the RGO surface. Figure S10b presents the high-resolution C 1s spectra. The peak at 284.8 eV corresponds to the C=C bond of the carbon framework. Notably, the characteristic peaks associated with oxygen-containing functional groups in the original RGO were significantly diminished or absent, indicating that the metal species were anchored through coordination interactions with surface oxygen-containing functional groups during the loading process. The O 1s peak deconvolution results shown in Figure S10c reveal a prominent peak at 530.4 eV on the Cu_6_Co_4_/RGO surface, corresponding to lattice oxygen [39]. This peak is more intense compared to that observed in Cu/RGO and Co/RGO, indicating a higher degree of metal oxidation. These findings are consistent with the HRTEM analysis, further confirming the presence of a greater distribution of metal oxides on the surface of Cu_6_Co_4_/RGO. The high-resolution Cu 2p XPS spectra are shown in Figure 2j. For Cu_6_Co_4_/RGO, the characteristic peaks at 932.8 eV and 934.6 eV correspond to the Cu^+1/0^ (2p^3/2^) and Cu^+2^ (2p^3/2^) states, respectively [39,42]. Similar peaks were observed for Cu/RGO at 934.9 eV and 933.1 eV. However, the binding energies of the Cu_6_Co_4_/RGO peaks are slightly shifted toward lower values compared to those of Cu/RGO. This shift can be attributed to electron transfer from Co to Cu at the bimetallic interface, indicating strong electronic interactions between the two metals [38,43]. At the same time, the intensity of the Cu^2+^ peak in Cu_6_Co_4_/RGO is higher than that in Cu/RGO, indicating a greater proportion of metal oxides present in the bimetallic catalyst. This further supports the formation of a multiphase interface composed of bimetallic oxides on the material surface. Auger electron spectroscopy (AES) was employed to distinguish the distribution of Cu^0^ and Cu^+^ species. As shown in Figure 2k, compared with Cu/RGO, the proportion of Cu^0^ decreased, while that of Cu^+^ increased, in Cu_6_Co_4_/RGO. Previous studies have shown that Cu_2_O/Cu active sites facilitate the adsorption and initial deoxygenation of NO_3_^−^. Therefore, the increased surface content of the Cu_2_O phase in Cu_6_Co_4_/RGO is favorable for NO_3_^−^ adsorption and reduction. The high-resolution Co 2p XPS fitting results are presented in Figure 2l. Compared with Co/RGO, the proportion of metallic Co^0^ on the Cu_6_Co_4_/RGO surface was significantly reduced. The main peaks at 780.5 eV and 782.5 eV correspond to CoO and Co_3_O_4_, respectively, and exhibit a shift toward higher binding energies compared to the Co/RGO peaks at 780.2 eV and 782.1 eV [44,45]. This shift further confirms electron transfer from Co to Cu at the heterogeneous interface of the multiphase metal oxides.

The metal content and chemical states on the surface of the Cu_6_Co_4_/RGO material were further investigated using thermogravimetric–differential scanning calorimetry (TG-DSC). As shown in Figure S11a, the DT curve for RGO indicates a gradual weight loss as the temperature increased from room temperature to 350 °C, eventually stabilizing. This weight loss can be attributed to the desorption of adsorbed water and the oxidative decomposition of surface oxygen-containing functional groups [14]. In comparison, the mass of Cu_6_Co_4_/RGO initially decreased to 97.72%, then slightly increased to 98.32% in the same temperature range. The initial decrease was due to the removal of surface-adsorbed water, while the subsequent mass gain can be attributed to the gradual oxidation of zero-valent metals present on the material surface in the air atmosphere. This oxidation behavior is further supported by the TEM and XPS characterization results. As the temperature continued to rise to 1000 °C, a significant mass loss occurred in the range 400–600 °C; this loss can be attributed to the rapid oxidation and decomposition of the carbon matrix in the air atmosphere. Based on the overall mass change, the estimated metal loading on the surface of Cu_6_Co_4_/RGO was approximately 12.42%. The derivative thermogravimetric (DTG) curves, obtained from the first-order derivative of the DT curves and shown in Figure S11b, reveal characteristic peaks for water desorption at 97.7 °C for RGO and 82.1 °C for Cu_6_Co_4_/RGO. The primary oxidation and decomposition peaks of the carbon matrix appeared at 529.8 °C and 411.4 °C, respectively. Additional peaks at 894.2 °C and 882.8 °C correspond to the further thermal degradation of the graphene structure in an oxygen-rich atmosphere. The differential scanning calorimetry (DSC) results are presented in Figure S11c. RGO exhibited an endothermic peak near 100 °C, corresponding to the volatilization of adsorbed water on its surface. In contrast, Cu_6_Co_4_/RGO displayed exothermic behavior at the same temperature, which can be attributed to the oxidation of metal species on the material surface. Pronounced exothermic peaks observed at 529.8 °C for RGO and 512.4 °C for Cu_6_Co_4_/RGO correspond to the thermal oxidation of carbon materials in each sample. The exothermic response beyond 850 °C was associated with the further oxidative decomposition of the graphene structure. Based on these material characterization results, it can be concluded that effective metal loading and anchoring can be achieved using RGO as a substrate. Oxygen-containing functional groups on the RGO surface served as coordination sites for metal ions, enabling metal immobilization through thermal reduction and annealing. The resulting composite multiphase system, consisting of Cu and Co, along with their respective oxides, formed the active catalytic sites for the ENRA reaction.

### 4.3. Performance Study of Catalysts for Nitrate Reduction to Ammonia

**Figure 3:**
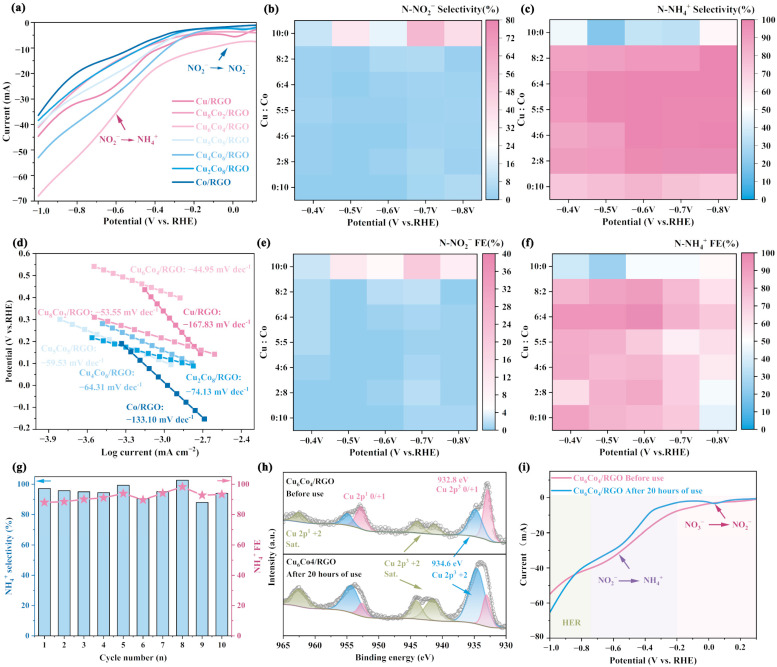
(**a**) LSV curves of different catalysts. (**b**) Heat map of N-NO_2_^−^ selectivity. (**c**) Heat map of N-NH_4_^+^ selectivity. (**d**) Tafel slope statistics for different materials. (**e**) Heat map of Faradaic efficiency (FE) for N-NO_2_^−^. (**f**) Heat map of Faradaic efficiency (FE) for N-NH_4_^+^. (**g**) Long-term electrolysis performance of Cu_6_Co_4_/RGO: selectivity and FE for ammonia production. (**h**) Analysis of copper valence states on the Cu_6_Co_4_/RGO surface before and after electrolysis. The gray circle represents the original test data, and the gray line represents the fitting analysis data. (**i**) LSV results for Cu_6_Co_4_/RGO before and after electrolysis.

Cyclic voltammetry (CV) and linear sweep voltammetry (LSV) tests were conducted in electrolytes both with and without nitrate nitrogen (NO_3_^−^). The results are presented in Figure S12a,b. In the presence of NO_3_^−^, a characteristic reduction peak corresponding to the conversion of NO_3_^−^ to NO_2_^−^ appeared in the range 0~−0.2 V and was followed by a second peak at −0.4~−0.7 V, corresponding to the reduction of NO_2_^−^ to NH_4_^+^ [46,47]. Notably, the intensity of the reduction peak in the −0.4~−0.7 V range initially increased and then decreased with increasing Co content in the bimetallic catalyst, reaching a maximum for Cu_6_Co_4_/RGO. The results for LSV in the presence of NO_3_^−^ exhibited a similar trend (Figure 3a). At −0.7 V, the reduction current of Cu_6_Co_4_/RGO reached 50.28 mA, which was 1.73 times higher than that of Cu/RGO (29.08 mA) and 2.96 times higher than that of Co/RGO (16.98 mA), demonstrating superior electrocatalytic performance. CV and LSV tests were also performed in the absence of NO_3_^−^ (Figure S12b,c) to assess the material’s activity in the hydrogen evolution reaction (HER). At −0.3 V, a characteristic peak attributed to hydrogen generation via water adsorption appeared, and with further increase in applied potential, the reduction current rose to 10–30 mA at −0.7 V [48]. These results indicate that HER activity intensified with increasing Co content, consistent with the intrinsic capacity of cobalt for hydrogen evolution.

Electrochemical impedance spectroscopy (EIS) and Tafel analysis were conducted to further evaluate the electrochemical performance of the samples [42]. As shown in Figure S12d, the Nyquist plots exhibit a typical semicircle in the high-frequency region and a straight line in the low-frequency region. The magnified view of the high-frequency region reveals that Cu_6_Co_4_/RGO exhibited the smallest charge-transfer resistance, indicating more efficient charge transport across the electrode surface. This suggests that Cu_6_Co_4_/RGO possesses superior electrocatalytic activity compared to the other samples. Tafel analysis is commonly used to evaluate the electrochemical kinetics of materials and can also provide insights into their corrosion resistance based on corrosion current and corrosion potential [39]. As shown in Figure S12e, Cu_6_Co_4_/RGO exhibited a lower corrosion current compared to Cu/RGO and Co/RGO, indicating superior corrosion resistance. The Tafel slope, calculated from the fitted cathodic Tafel curve (Figure 3d), was lowest for Cu_6_Co_4_/RGO, at 44.95 mV/dec. This suggests that the material facilitated rapid charge transfer and efficient mass transport at the electrode surface. These results of electrochemical characterization consistently demonstrated that Cu_6_Co_4_/RGO exhibited the best electrochemical kinetic performance among the tested catalysts.

The electrocatalytic performance of different materials under various applied voltages was evaluated using constant-voltage chronoamperometry. All electrolysis experiments were conducted for a fixed duration of 2 h in an electrolyte composed of 0.01 mol/L NaNO_3_ + 0.1 mol/L Na_2_SO_4_. Following electrolysis, the concentrations of reaction products in the solution were determined using UV-Vis spectroscopy, and the corresponding component distributions were calculated based on standard calibration curves, as illustrated in Figures S13 and S14. It was observed that the nitrate-reduction capability of the metal-loaded materials was significantly enhanced. In all samples, the removal rate of NO_3_^−^ showed a positive correlation with the applied reduction voltage: the higher the applied voltage, the greater the nitrate removal efficiency. Moreover, the electrocatalytic performance varied with the Cu–Co loading ratio. As the Cu–Co ratio decreased, the nitrate removal rate initially increased and then declined. Additionally, both the product selectivity and Faradaic efficiency (FE) were influenced by the Cu–Co composition, indicating that the metal ratio plays a critical role in determining the catalytic performance.

During the reduction of NO_3_^−^ to NH_4_^+^, water adsorption and dissociation also occur on the catalyst surface, generating *H species. These *H species are essential for the hydrogenation steps leading to NH_4_^+^ formation. However, an excessive accumulation of *H can promote the competing *H-*H hydrogen evolution reaction (HER), thereby decreasing the Faradaic efficiency. Therefore, achieving a balance between the generation and consumption rates of *H is considered a key challenge in improving the efficiency of electrocatalytic ammonia production [10,49]. The intrinsic hydrogen-adsorption capacity of the catalyst can be enhanced by increasing the Co content in the material, while the generation and consumption of *H species can be further regulated by adjusting the applied reduction voltage [11,41]. To further investigate the influence of metal composition and applied voltage on performance in ammonia production, the selectivity and Faradaic efficiency (FE) for NO_2_^−^ and NH_4_^+^ were calculated based on the product composition in the electrolyte. The results are presented in Figure 3b,c,e,f. Overall, the introduction of Co into the catalyst significantly reduced both the selectivity and FE for the NO_2_^−^ intermediate, while enhancing the selectivity and FE for the target product NH_4_^+^ compared to the pure Cu catalyst. This indicates that the incorporation of Co improves the material’s intrinsic capacity for *H supply, thereby enhancing its performance in ammonia production. Additionally, increasing the reduction voltage led to a decrease in both NO_2_^−^ selectivity and FE, while NH_4_^+^ selectivity increased. Notably, both the Co-loading ratio and the applied voltage exhibited a volcano-type relationship with performance in ammonia production. At a constant voltage, an excessively high Co content led to decreases in both NH_4_^+^ selectivity and FE. Conversely, under a fixed Co loading ratio, excessively high reduction voltages resulted in increased NH_4_^+^ selectivity but decreased FE, suggesting that an optimal balance between composition and voltage is critical for maximizing catalytic efficiency. These observations indicate that a dynamic balance must be achieved between the rate of *H consumption by nitrogen and oxygen species and the rate of *H generation via H_2_O dissociation. Such a balance is essential to ensure high selectivity in ammonia production while maintaining a high FE. An insufficient supply of *H leads to the accumulation of NO_2_^−^ intermediates, whereas an excessive supply promotes the competing hydrogen-evolution reaction (HER), thereby reducing the efficiency of ammonia synthesis. Based on comprehensive experimental results, a Cu:Co loading ratio of approximately 6:4 delivers the optimal overall performance across a range of applied voltages. At −0.6 V, this composition achieves an NH_4_^+^ selectivity of 99.86% and a FE of 96.54%, highlighting its excellent electrocatalytic activity for ammonia production. The statistical analysis of ammonia yield and TON indicates that Cu_6_Co_4_/RGO exhibits the highest performance, achieving an ammonia yield of 24.9 mmol g^−1^ h^−1^ at −0.6 V and demonstrating the optimal TON (Figure S15). The material exhibits excellent catalytic performance compared to other materials (Table S5) [25,50,51,52,53,54,55,56,57,58].

The stability of the catalyst was evaluated using long-term constant-voltage electrolysis. As shown in Figure S16, after 10 cycles of continuous operation, the NO_3_^−^-removal ratio remained above 90%. Meanwhile, the selectivity for NO_2_^−^ intermediate byproducts was below 5%, and its Faradaic efficiency gradually decreased with increasing cycle number, ultimately stabilizing at less than 3%. Throughout the electrolysis process, the selectivity for the target product, NH_4_^+^, remained consistently above 95% and the Faradaic efficiency for ammonia production exceeded 90% (Figure 3g). These results indicate that the metal active sites remained firmly anchored to the carbon substrate, maintaining catalytic activity even after extended operation.

X-ray photoelectron spectroscopy (XPS) was employed to further investigate the changes in surface elemental states of Cu_6_Co_4_/RGO before and after electrolysis. As shown in the full survey spectra (Figure S17a), the surface elemental composition includes Cu, Co, O, and C. However, the intensity of the O 1s peak increased after electrolysis, indicating surface oxidation. This can be attributed to the oxidation of surface metal species upon exposure to air following contact with the electrolyte. To assess potential elemental leaching, ICP analysis was performed to determine the Cu/Co atomic ratio before and after 20 h of electrolysis. As shown in Table S4, the Cu/Co ratio decreased slightly, from 1.46 to 1.43, corresponding to a reduction of 2.05% and suggesting minimal metal dissolution during the electrochemical process. The Cu 2p spectrum is shown in Figure 3h. Compared to the state before electrolysis, the intensity of the Cu^0/+1^ peak at 932.8 eV was reduced, while the Cu^+2^ peak at 934.6 eV was significantly enhanced [39]. Additionally, the satellite peaks associated with Cu^+2^ in the range of 940–947 eV were more pronounced, indicating an increased surface concentration of Cu^+2^ species after electrolysis. AES (Figure S17b) further revealed that the Cu_2_O content decreased while the CuO content increased after electrolysis, supporting the XPS findings. The high-resolution Co 2p spectrum is shown in Figure S17c. The Co^0^ peak at 778.85 eV decreased in intensity, and the main peak for CoO at 780.3 eV also weakened. In contrast, the peak for Co_3_O_4_ at 782.2 eV became more intense, indicating a higher proportion of high-valence cobalt oxides [42]. These changes can be attributed to the oxidation of surface metal species upon exposure to oxygen in air after electrolysis.

The electrochemical performance of Cu_6_Co_4_/RGO before and after electrolysis was further evaluated. Following long-term cycling, the sample was subjected to CV testing. The first CV cycle provided insights into the relationship between changes in surface composition and the applied potential. As shown in Figure S18a, a reduction peak corresponding to the conversion of CuO to Cu_2_O appeared in the range of 0~−0.2 V for the post-electrolysis sample, with a higher intensity than that observed prior to electrolysis [47,59]. This observation is consistent with the XPS results, confirming that surface metal oxidation occurred due to air exposure after electrolysis. In the last cycle CV test (Figure S18b), the reduction current of the sample remained comparable to that before electrolysis, indicating that the electrochemical activity was largely retained. After stabilization through CV activation, LSV was performed to evaluate the reduction current of the material. As shown in Figure 3i, the CuO reduction peak at 0~−0.2 V remained nearly unchanged compared to the pre-electrolysis sample. However, in the voltage range relevant to the ENRA reaction, the post-electrolysis sample exhibited a reduced current response. Specifically, at −0.6 V, the reduction peak current recovered to 88.72% of its initial value prior to electrolysis. These results demonstrate that Cu_6_Co_4_/RGO possesses excellent electrochemical stability, maintaining a FE for ammonia production above 90% even after 20 h of continuous operation. Although some oxidation of surface metal active sites occurred due to air exposure, there was no significant metal dissolution. Notably, the oxidized sites could be effectively reduced during CV activation and the catalytic activity could be restored to 88.72% of its original level, confirming the material’s robust reusability and stability.

### 4.4. Mechanism of Electro Denitrification

**Figure 4:**
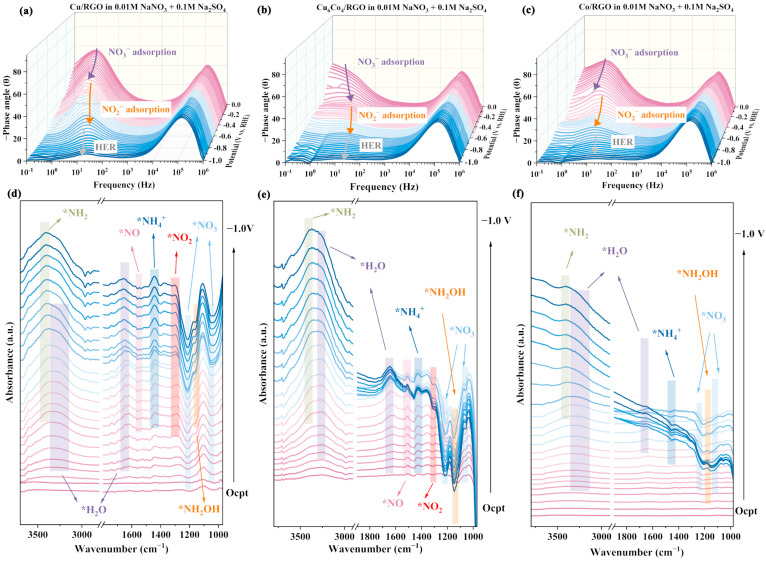
In situ electrochemical EIS Bode plots in 0.01 mol/L NaNO_3_ + 0.1 mol/L Na_2_SO_4_ electrolyte: (**a**) Cu/RGO, (**b**) Cu_6_Co_4_/RGO, (**c**) Co/RGO. In situ FTIR spectra: (**d**) Cu/RGO, (**e**) Cu_6_Co_4_/RGO, (**f**) Co/RGO. The symbol * in the figure represents the adsorption state.

The electrochemical reaction kinetics of Cu/RGO, Cu_6_Co_4_/RGO, and Co/RGO were investigated using in situ EIS, and the results are presented in Figure 4a–c. The frequency position and corresponding phase angle of the peaks are commonly used to evaluate reaction kinetics. A higher peak frequency indicates a higher electrochemical reaction rate, where the peak intensity is associated with reaction resistance and stronger peaks imply greater kinetic resistance [60]. Compared to Cu/RGO and Co/RGO, the Cu_6_Co_4_/RGO catalyst exhibited a higher peak frequency, indicating more rapid electron transfer and faster reaction kinetics. Additionally, the lower peak intensity at the phase angle suggests reduced electrochemical resistance during catalysis. These results collectively demonstrate that Cu_6_Co_4_/RGO possesses superior electrocatalytic kinetic properties.

In situ FTIR was employed to monitor the reaction process and elucidate the reaction pathway, as shown in Figure 4d–f. The N–H stretching vibration peaks were observed at 3270 cm^−1^ and 1425 cm^−1^. Absorption peaks at 1152 cm^−1^ and 1053 cm^−1^ correspond to the stretching and bending vibrations of the N–O bond in *NO_3_ species [37,61]. The characteristic peak at 1210 cm^−1^ can be attributed to the symmetric and asymmetric stretching vibrations of *NO_2_ [62]. The peak at 1540 cm^−1^ is associated with the bridging N–O vibration of *NO [63]. A key intermediate for ammonia formation, *NH_2_OH, was identified by a peak at 1180 cm^−1^. Additionally, absorption bands near 1650 cm^−1^ and 3200 cm^−1^ correspond to the bending and stretching vibrations of O–H bonds from adsorbed H_2_O [10]. As shown on the surface of Cu/RGO (Figure 4c), with increasing reduction voltage, the intensity of the *NO_3_ adsorption peak gradually decreased, indicating an accelerated reduction of *NO_3_ at higher voltages. However, the *NO_2_ adsorption peak concurrently increased, suggesting the accumulation of *NO_2_ intermediates on the catalyst surface. Meanwhile, the weak H_2_O adsorption peak indicates that Cu/RGO exhibited limited ability to adsorb and dissociate water to generate *H species, thereby constraining the further reduction and conversion of *NO_2_ intermediates. On the surface of Cu_6_Co_4_/RGO, the adsorption peak intensities of *NO_3_, *NO_2_, and other nitrogen oxide species gradually decreased with increasing reduction voltage and no accumulation of intermediates was observed. This indicates that Cu_6_Co_4_/RGO exhibited the capacity for faster reduction of nitrogen and oxygen species compared to Cu/RGO, particularly in facilitating the rapid deoxygenation of intermediates such as *NO_2_. According to previous studies, *H plays an essential role in the further reduction of intermediates like *NO_2_. Compared with Cu/RGO, Cu_6_Co_4_/RGO displayed a significantly stronger H_2_O adsorption peak, suggesting that the incorporation of Co enhanced the material’s ability to adsorb and dissociate water. This, in turn, improved *H supply efficiency, enabling rapid deoxygenation and boosting the overall efficiency of the ENRA reaction. On the surface of Co/RGO, the adsorption peak intensity of species such as *NO_3_ was significantly lower than those observed for Cu/RGO and Cu_6_Co_4_/RGO, indicating that the poor catalytic performance of Co/RGO was primarily due to its insufficient adsorption capacity for *NO_3_ target molecules. In contrast, Cu_6_Co_4_/RGO exhibited strong adsorption toward *NO_3_, along with an efficient *H supply, thereby enhancing the overall catalytic efficiency of the ENRA process. Based on the experimental results, the proposed nitrate-reduction pathway on Cu_6_Co_4_/RGO is as follows: NO_3_^−^ → *NO_3_ → *NO_2_ → *NO → *NOH → *NH_2_OH → *NH_2_ → *NH_3_ → NH_3_.

## 5. Conclusions

In conclusion, the surface functional groups of graphene oxide (GO) were modified via alkaline hydrothermal treatment, during which hydroxyl and epoxy groups were gradually converted into carbonyl and carboxyl groups. Using the modified GO as a support, a series of Cu–Co/RGO catalysts with varying Cu/Co ratios were successfully synthesized. Comprehensive material characterization confirmed that Cu and Co, in both metallic and oxide forms, were uniformly dispersed on the RGO surface, resulting in highly distributed catalytic active centers. Among them, the Cu_6_Co_4_/RGO catalyst exhibited the best electrocatalytic performance, achieving a nitrate-to-ammonia selectivity of 99.86% and a Faradaic efficiency of 96.54% at −0.6 V. Moreover, the catalyst showed excellent cycling stability, maintaining a Faradaic efficiency above 90% after 20 h of continuous operation, with negligible metal leaching. Even after electrochemical activation, the catalyst remained effective. In situ infrared spectroscopy revealed that the introduction of Co significantly enhanced water dissociation for hydrogen generation, improved intermediate adsorption behavior, suppressed intermediate accumulation, and facilitated the rapid conversion of nitrogen–oxygen species.

## Data Availability

The original contributions presented in this study are included in the article/Supplementary Material. Further inquiries can be directed to the corresponding author.

## Supplementary Materials

The following supporting information can be downloaded at: https://www.mdpi.com/article/10.3390/ma18112495/s1, The Supporting Information is freely available. In addition, it contains a number of graphs that support analysis.
