# Supplementary material for: Graphene Oxide-Anchored Cu–Co Catalysts for Efficient Electrochemical Nitrate Reduction
Source: Materials (Basel). 2025 May 26;18(11):2495. doi: 10.3390/ma18112495 (PMC12156831; doi:10.3390/ma18112495)
Supplement: Supplementary file 1 [file materials-18-02495-s001.zip › materials-3622522-supplementary.pdf]

Graphene Oxide-Anchored Cu-Co Catalysts for Efficient Electrochemical Nitrate Reduction

Haosheng Lan [a], Yi Zhang [a], Le Ding [a], Xin Li [a], Zhanhong Zhao [a], Yansen Qu [a], Yingjie Xia [a], Xinghua Chang [a b*]

[a] *School of Minerals Processing and Bioengineering, Central South University, Changsha 410083, China.*

[b] *Key Laboratory for Mineral Materials and Application of Hunan Province, School of Minerals Processing and Bioengineering, Central South University, Changsha 410083, China; Email:* changxinghua@csu.edu.cn

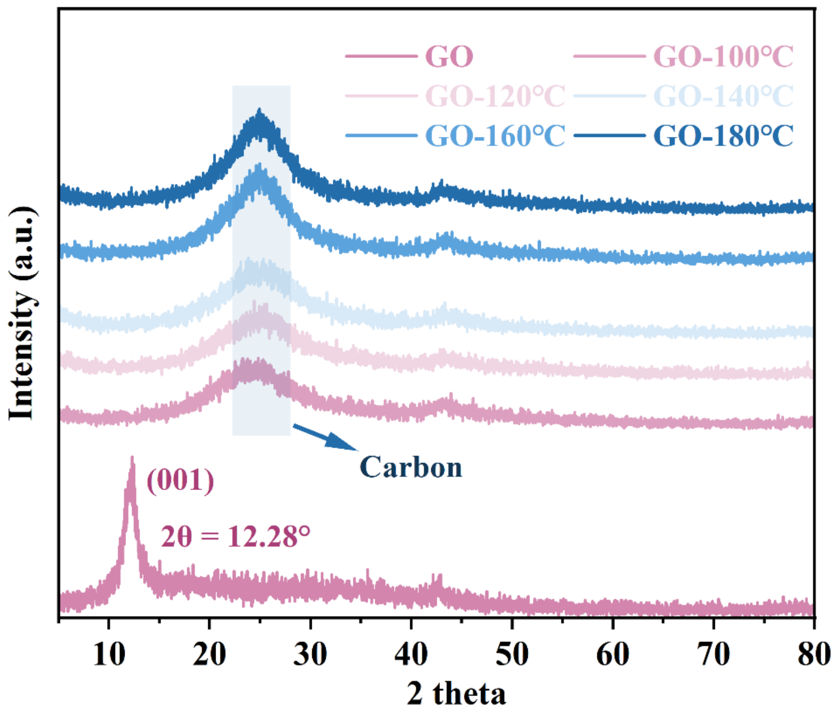

Figure S1. XRD results of graphene oxide after hydrothermal treatment at different temperatures

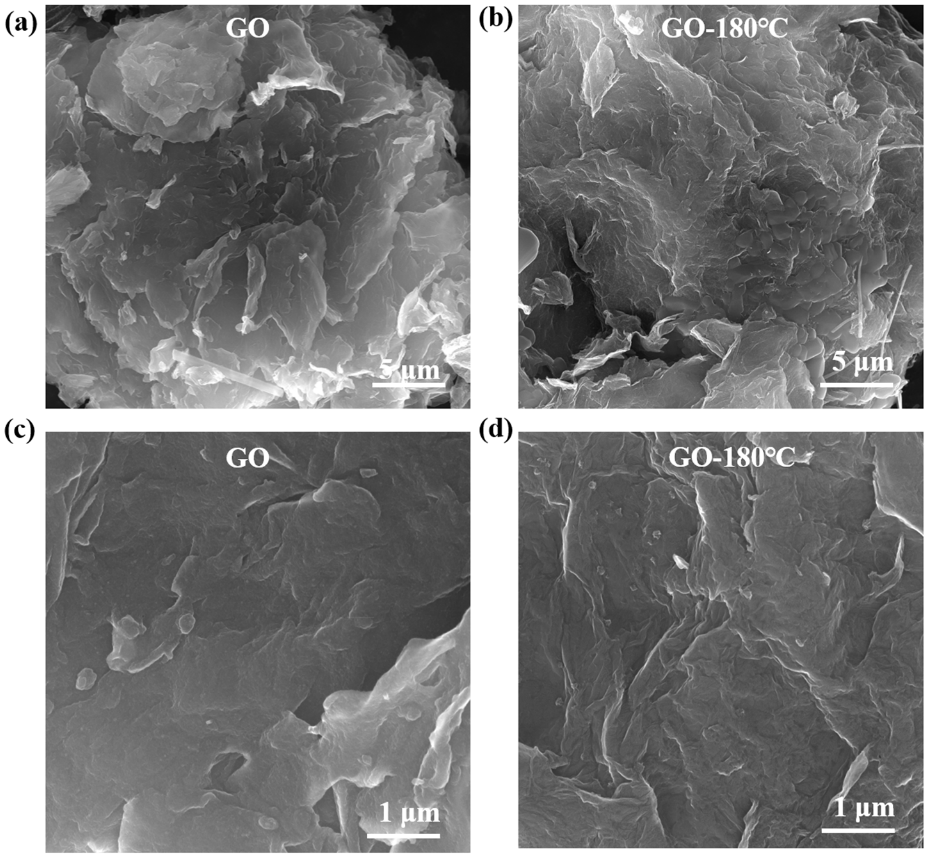

Figure S2. SEM results of GO and GO-180°C (a) (c) SEM of GO at 10000 and 100000 magnification. (b) (d) SEM image of GO-180°C at 10000 and 100000 magnification

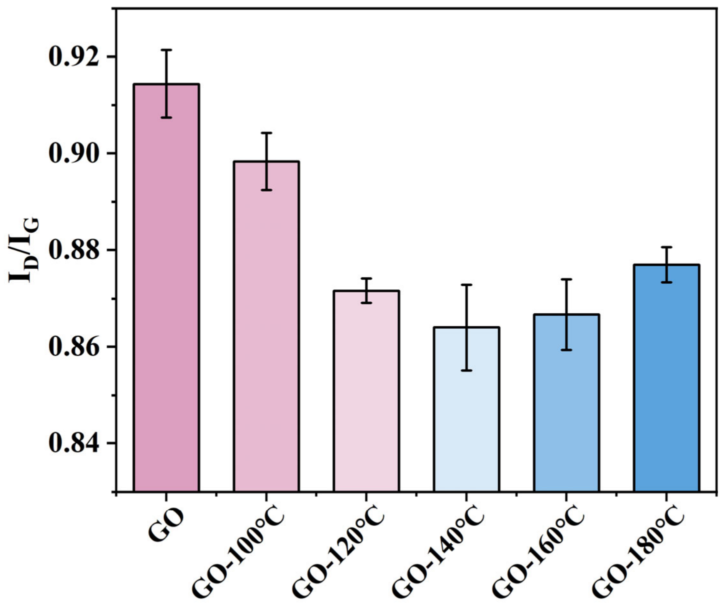

Figure S3. Statistical analysis of I_D/I_G values obtained from Raman measurements.

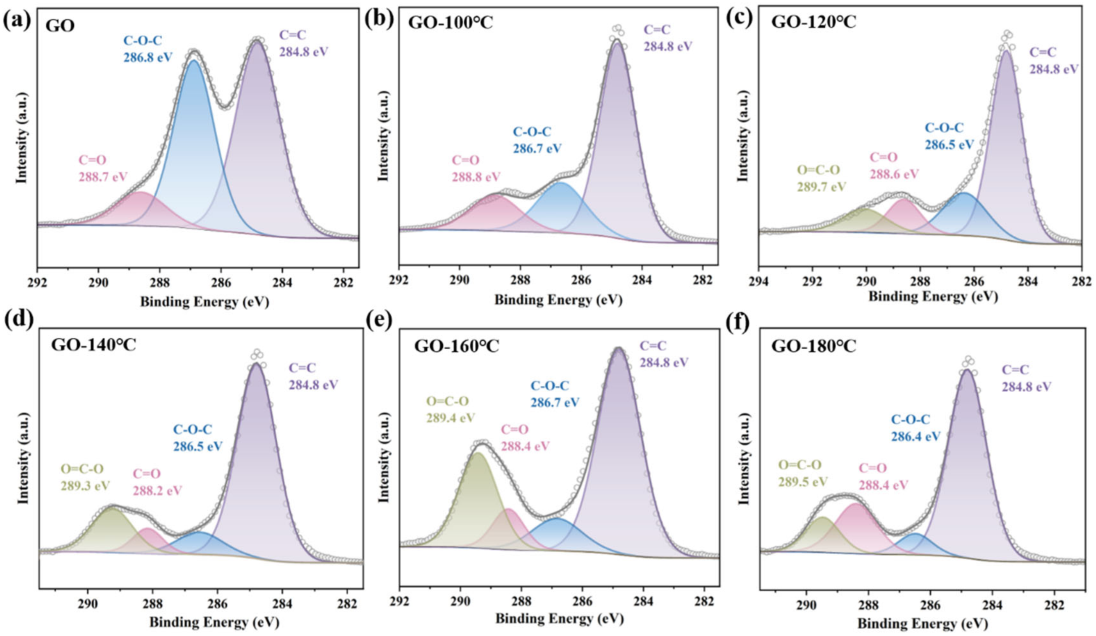

Figure S4. The peak fitting spectra of C1s after hydrothermal treatment of GO at different temperatures (a) GO. (b) GO-100℃. (c) GO-120℃. (d) GO-140℃. (e) GO-160℃. (f) GO-180℃.

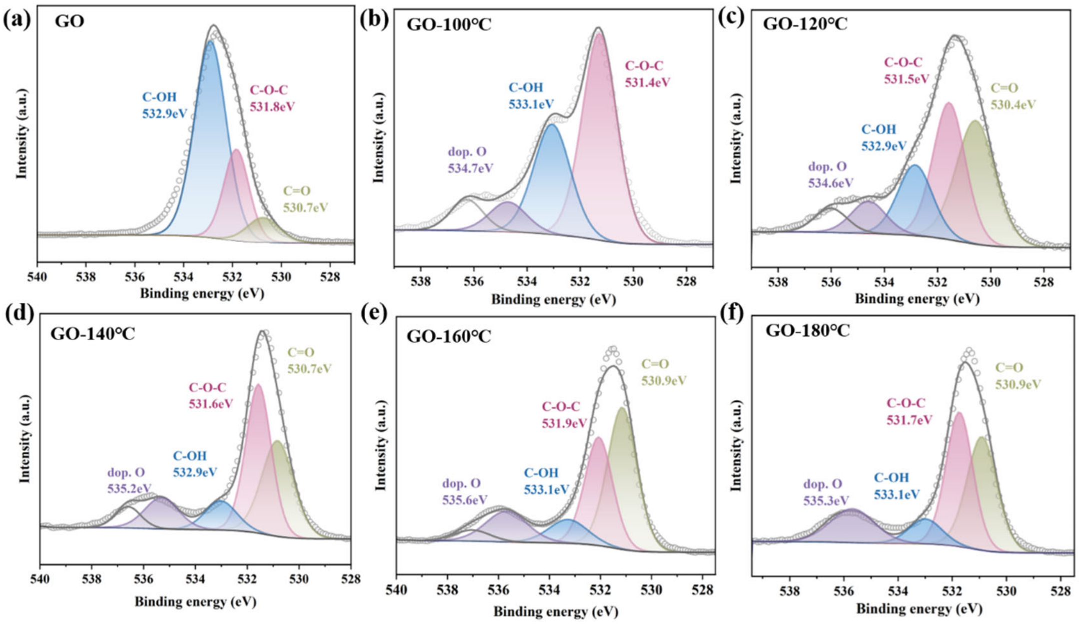

Figure S5. The peak fitting spectra of O1s after hydrothermal treatment of GO at different temperatures (a) GO. (b) GO-100℃. (c) GO-120℃. (d) GO-140℃. (e) GO-160℃. (f) GO-180℃.

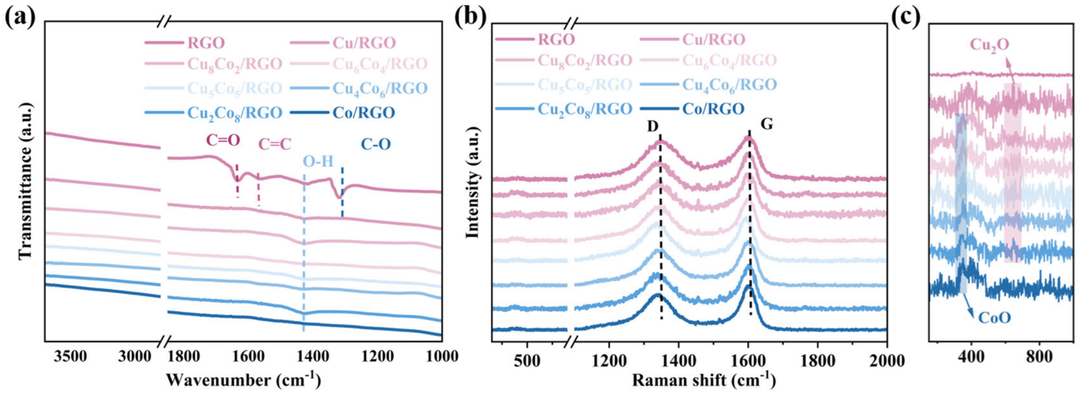

Figure S6. RGO loaded with different Cu/Co ratios (a) FTIR test result. (b) Raman detection results (c) The Raman spectrum is locally enlarged in the range of 190-1000cm^{-1}

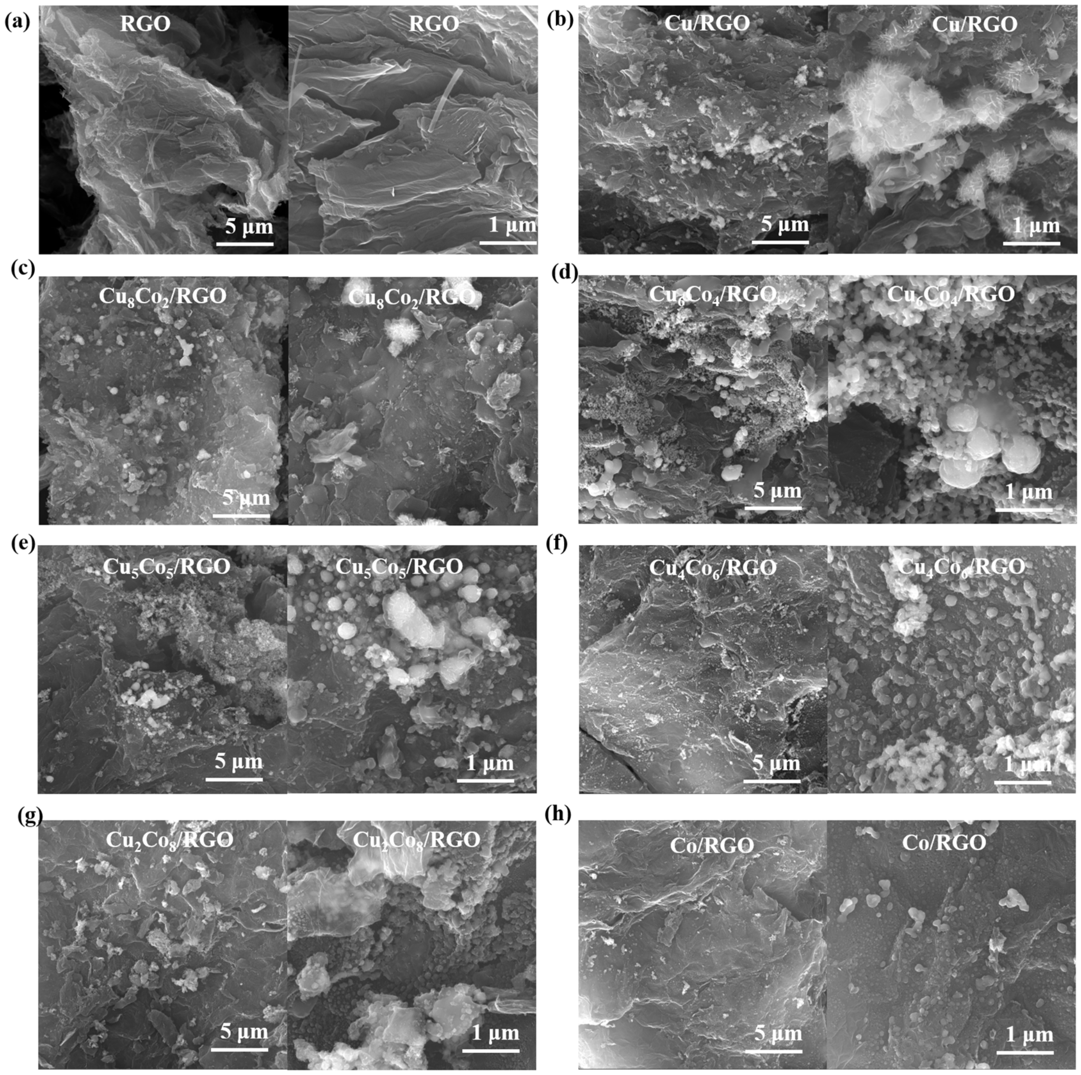

Figure S7. SEM images of different samples. (a)RGO. (b)Cu/RGO. (c)Cu_8Co_2/RGO. (d)Cu_6Co_4/RGO. (e)Cu_5Co_5/RGO. (f)Cu_4Co_6/RGO. (g)Cu_2Co_8/RGO. (h)Co/RGO.

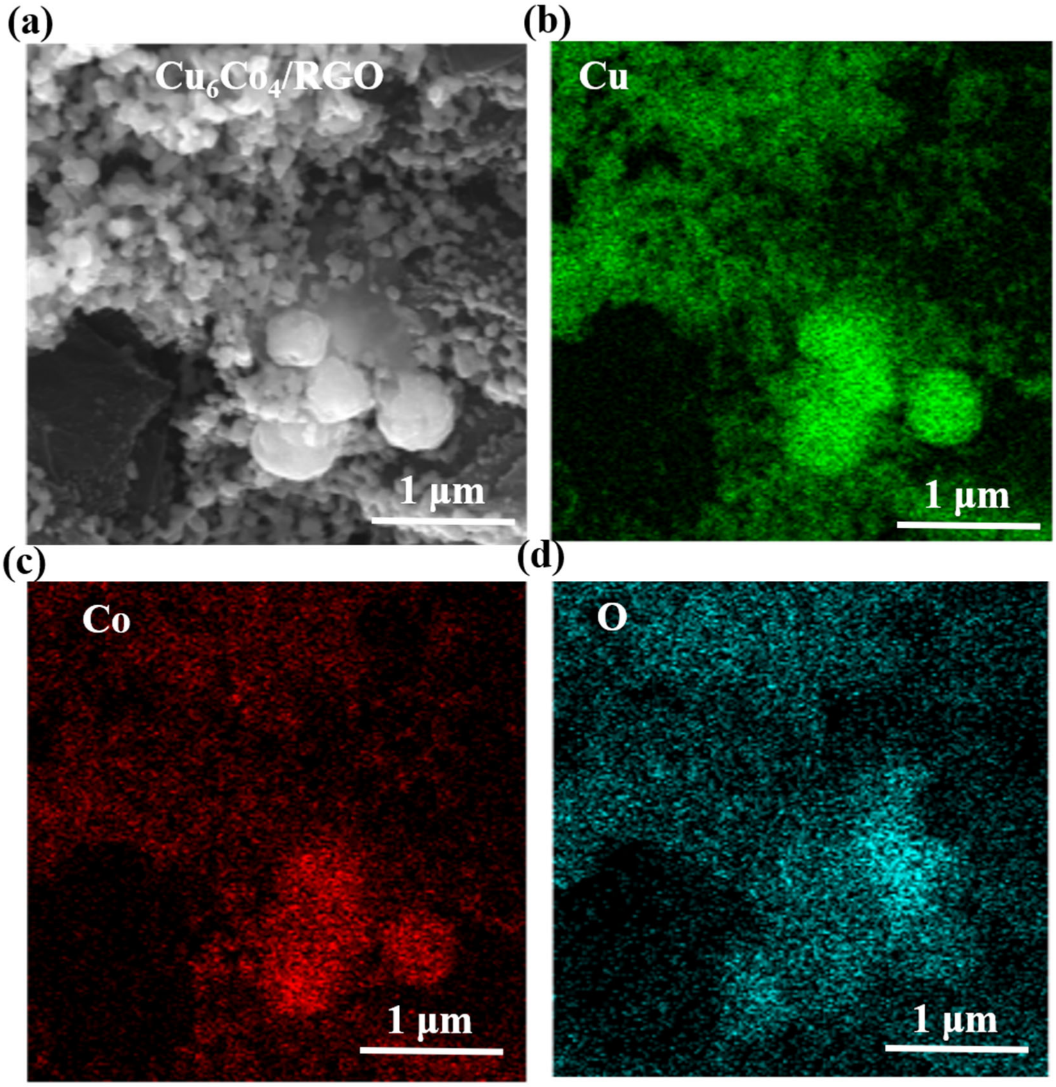

Figure S8. Cu_6Co_4/RGO morphology and element distribution diagram.

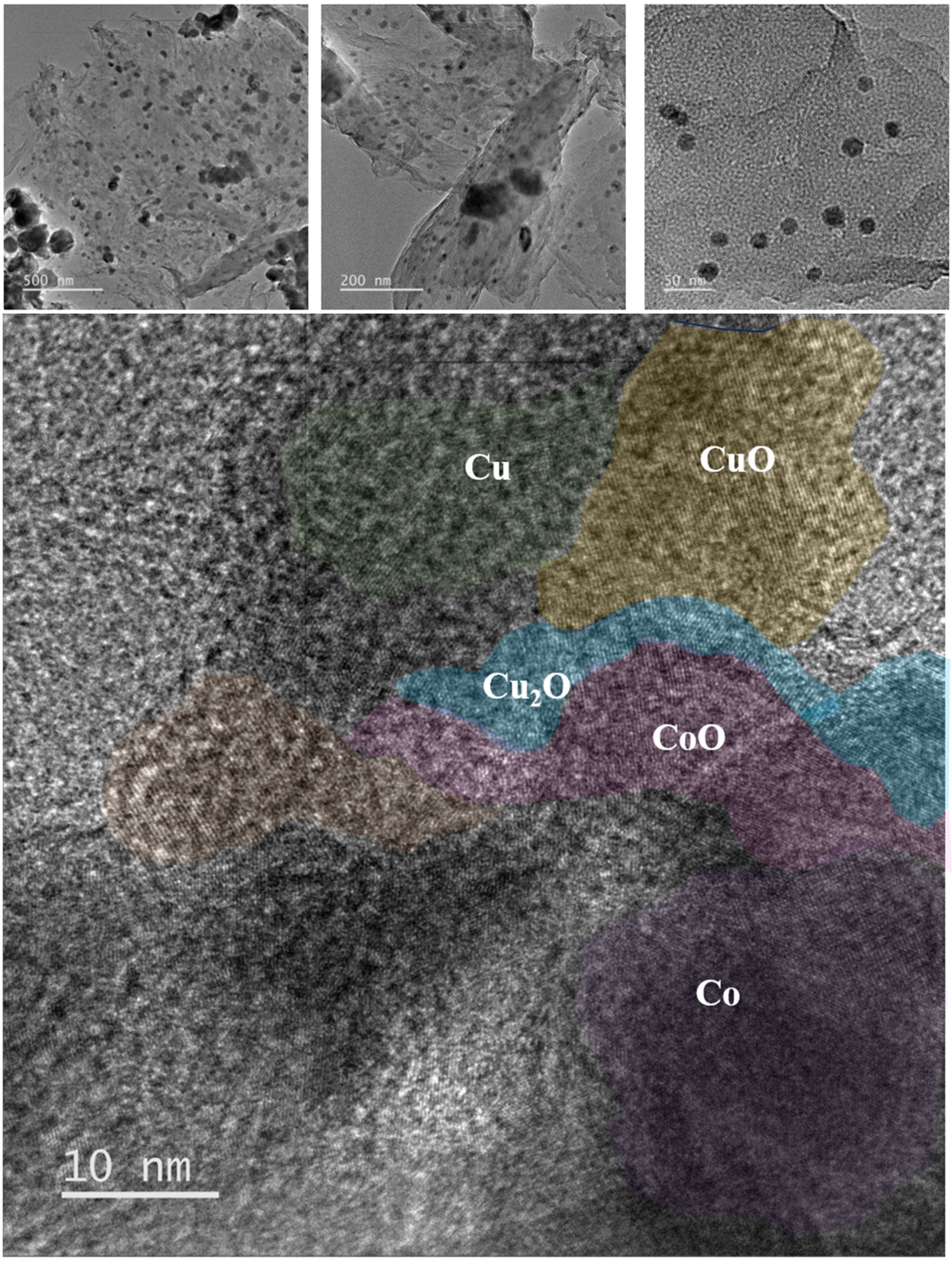

Figure S9 Cu_6Co_4/RGO TEM phase distribution results.

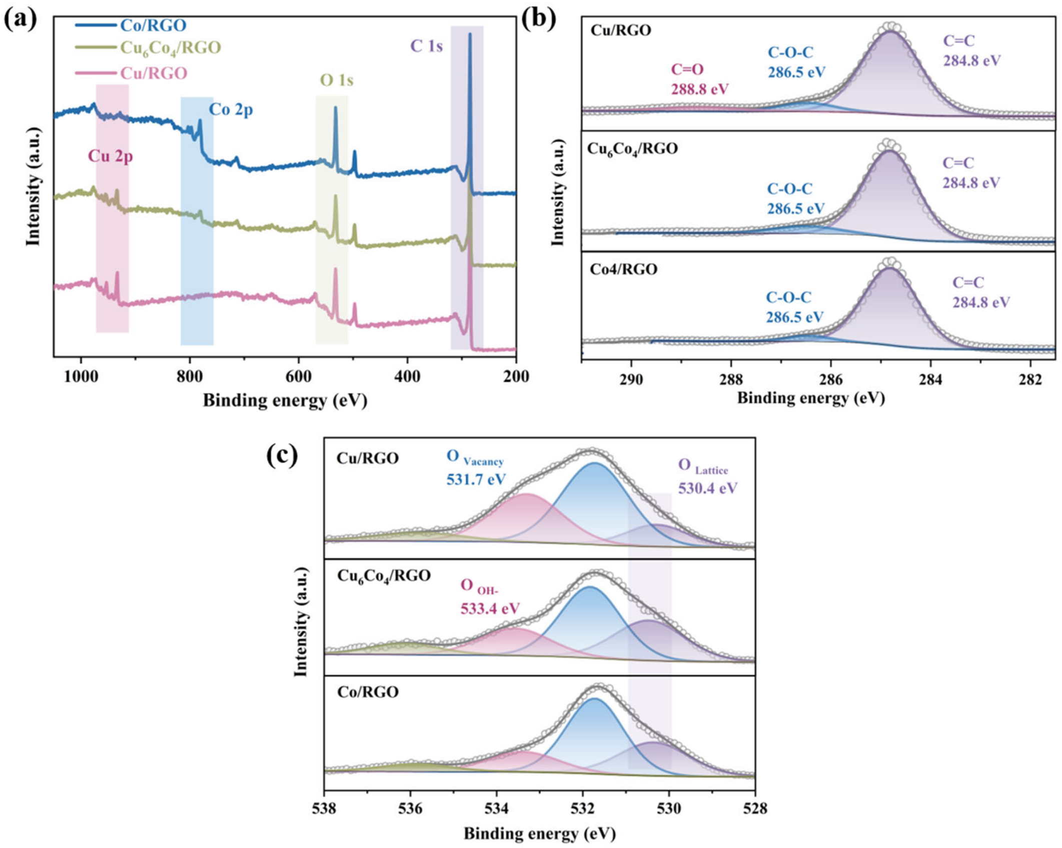

Figure S10. XPS results of Cu/RGO, Cu_6Co_4/RGO and Co/RGO. (a) Full spectrum detection. (b) Fitting results of C 1s fine spectral peaks (c) O 1s fine spectrum fitting peak splitting results.

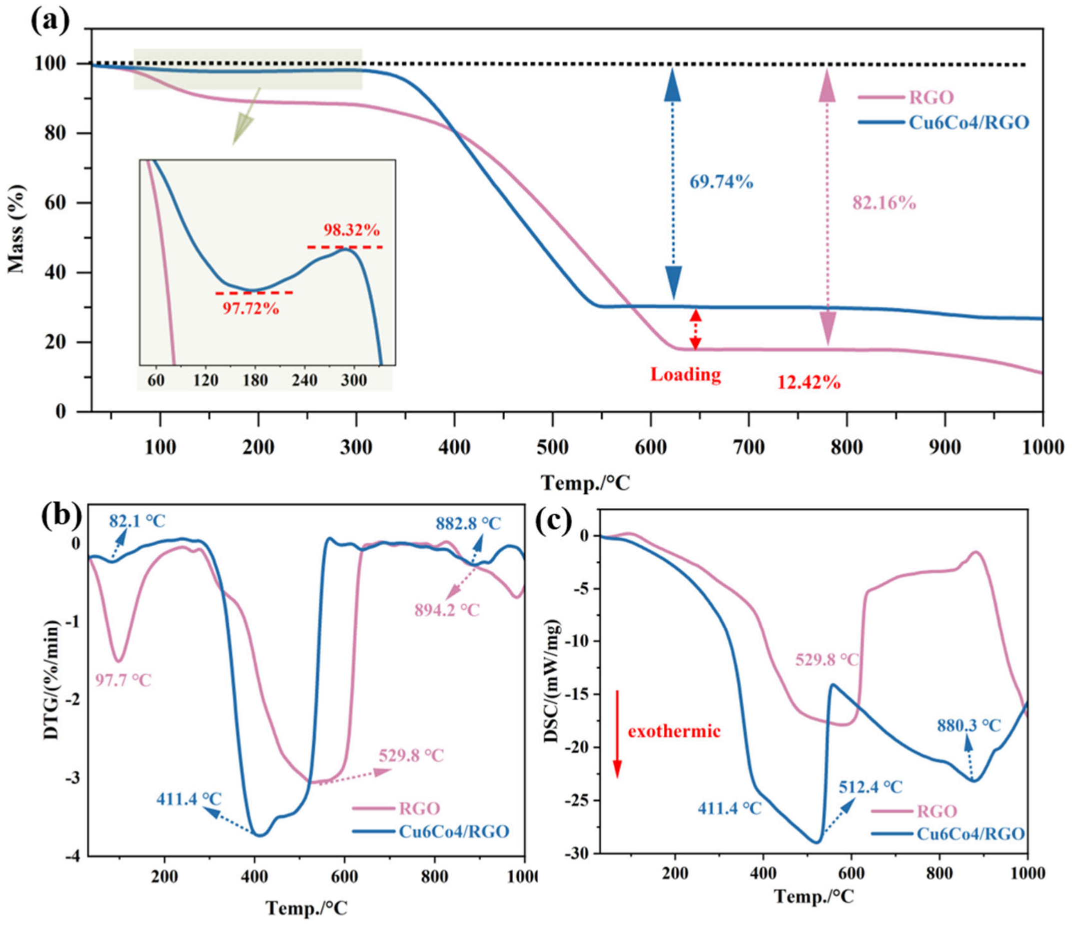

Figure S11. Analysis results of Cu_6Co_4/RGO thermogravimetric differential scanning test. (a) DT test result chart. (b) DTG analysis result chart. (c) DSC analysis result chart

It is worth noting that the thermal events of Cu_6Co_4/RGO occur at lower temperatures compared to those of pristine RGO. This shift can be attributed to the presence of metal particles, which act as thermal conductors after being loaded onto the RGO surface. These particles may facilitate localized temperature increases, thereby accelerating the decomposition or deoxygenation of oxygen-containing functional groups in graphene oxide and leading to earlier onset of weight loss. Additionally, the incorporation of metal particles may weaken the interlayer adhesion of the graphene sheets, making the pyrolysis of graphene oxide more facile and resulting in its earlier degradation.

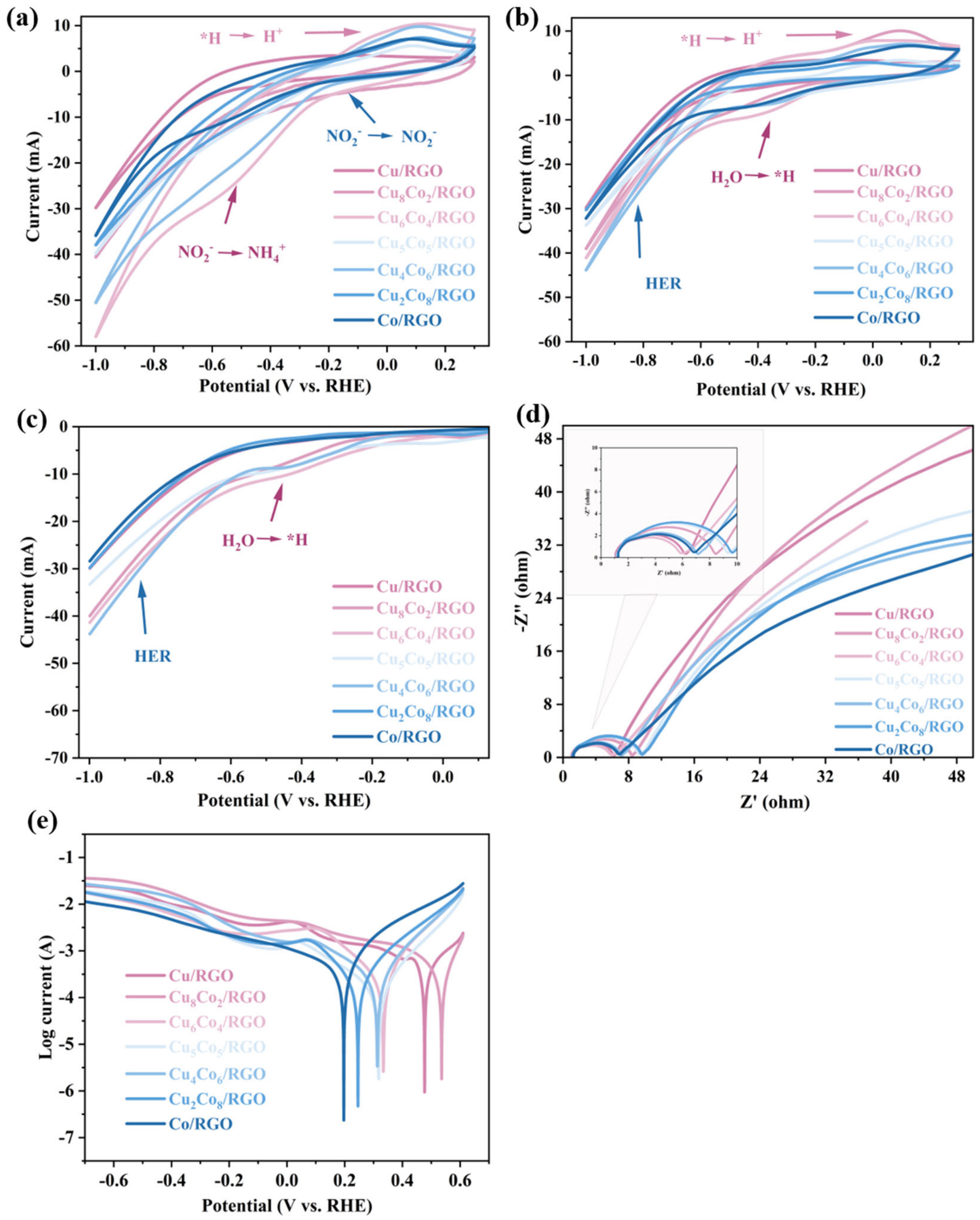

Figure S12. Electrochemical performance test results of different materials. (a) CV test results in electrolyte containing N-NO_3^-. (b) (c) CV and LSV test results in electrolyte without N-NO_3^-. (d) EIS test Nuquist diagram, the illustration is high frequency Nuquist enlarged diagram. (e) Tafel test curve.

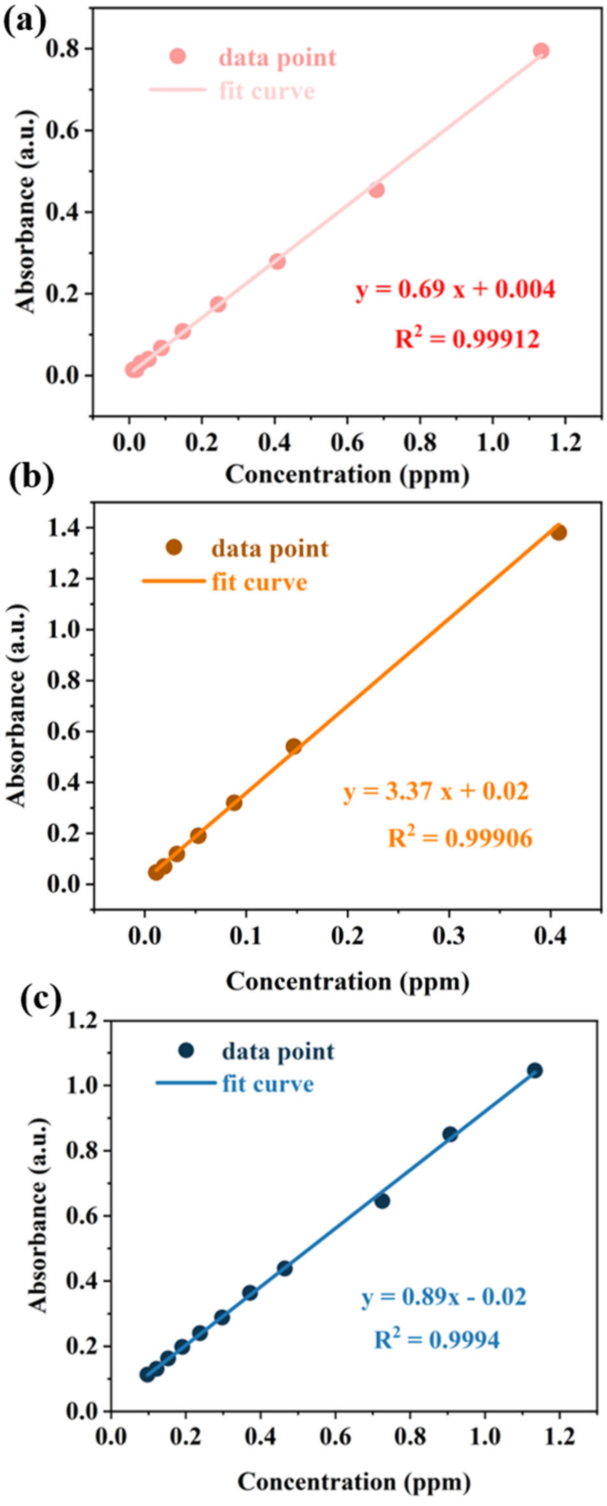

Figure S13. Standard curve of UV spectrum test. (a) NO_3^-. (b) NO_2^-. (c) NH_4^+.

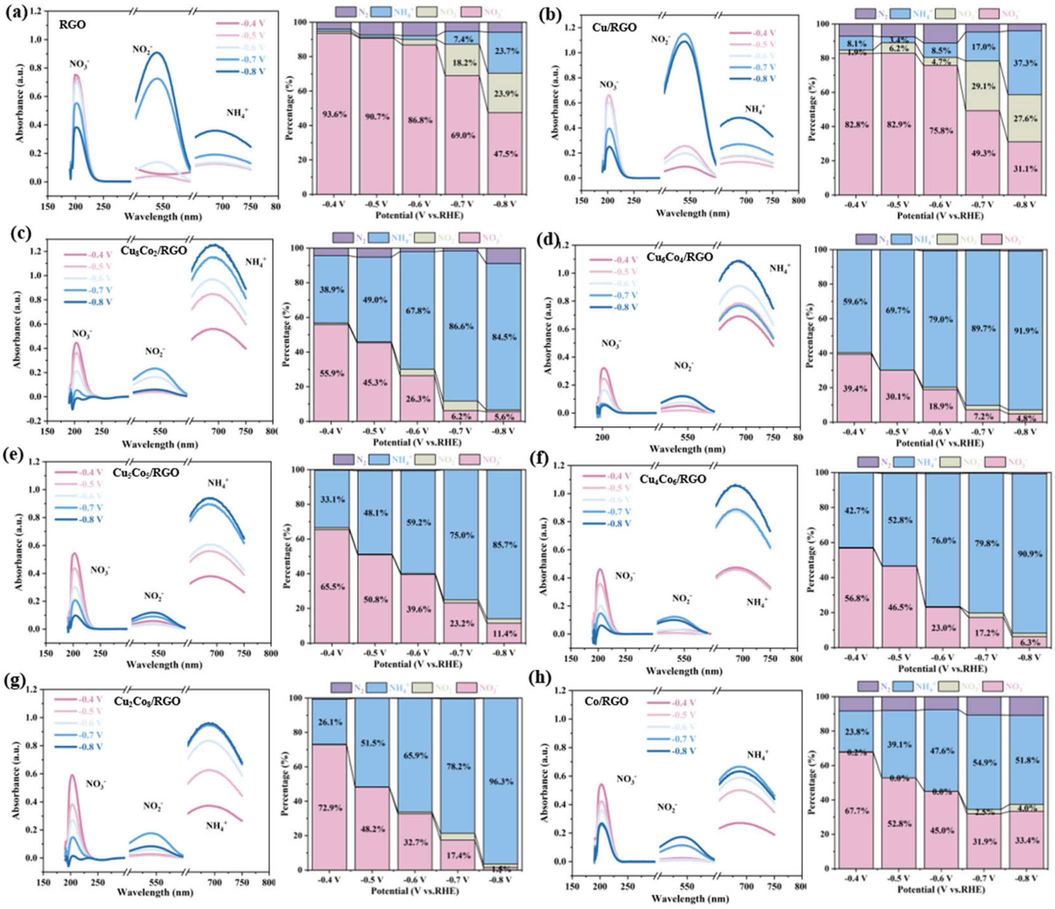

Figure S14. UV-Vis spectra and corresponding product distribution in the electrolyte after electrolysis for different materials. (a)RGO. (b)Cu/RGO. (c)Cu_8Co_2/RGO. (d)Cu_6Co_4/RGO. (e)Cu_5Co_5/RGO. (f)Cu_4Co_6/RGO. (g)Cu_2Co_6/RGO. (h)Co/RGO.

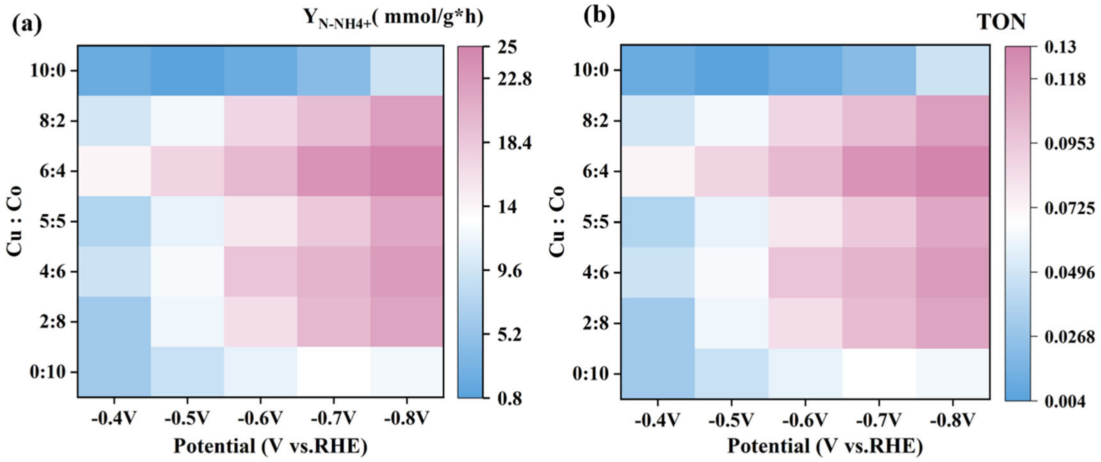

Figure S15 (a) Ammonia yield and (b) TON statistical results of the materials.

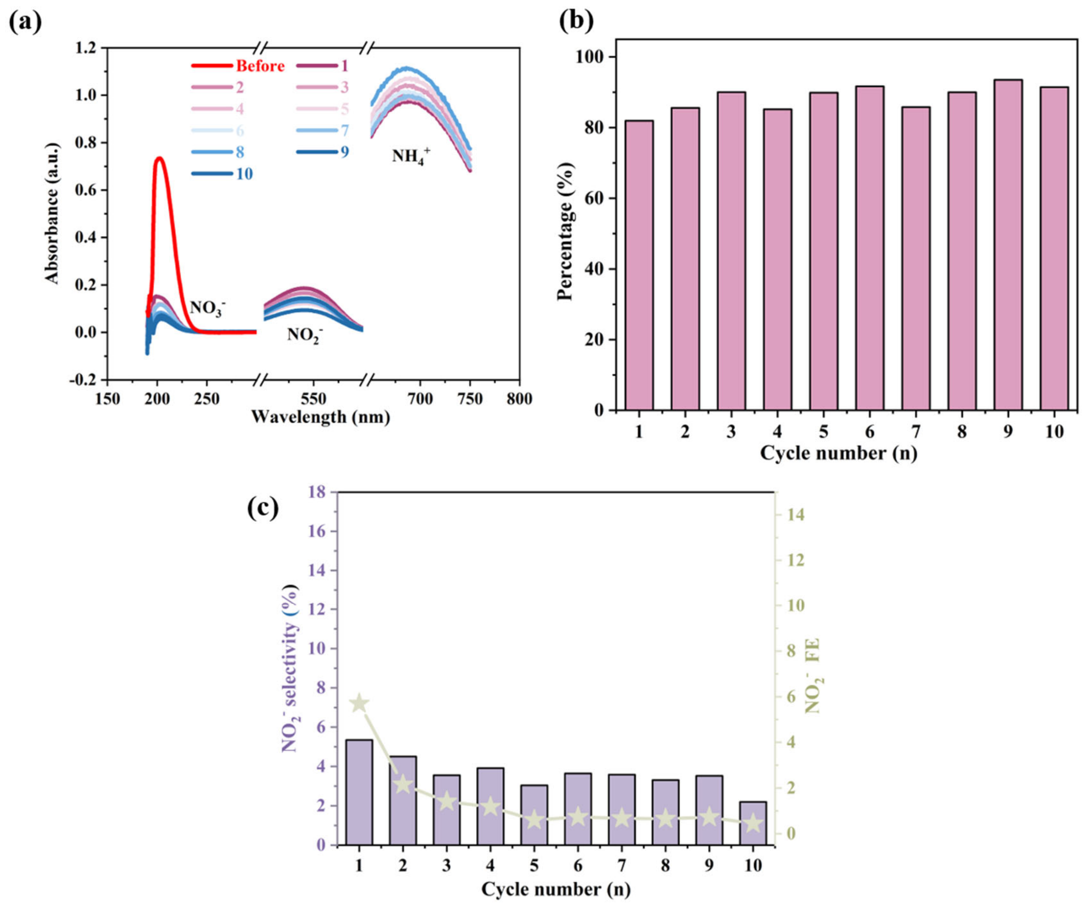

Figure S16. (a) UV-Vis spectra of Cu_6Co_4/RGO after long-term electrolysis. (b) NO_3^- removal ratio. (c) Selectivity and Faradaic efficiency (FE) for NO_2^-.

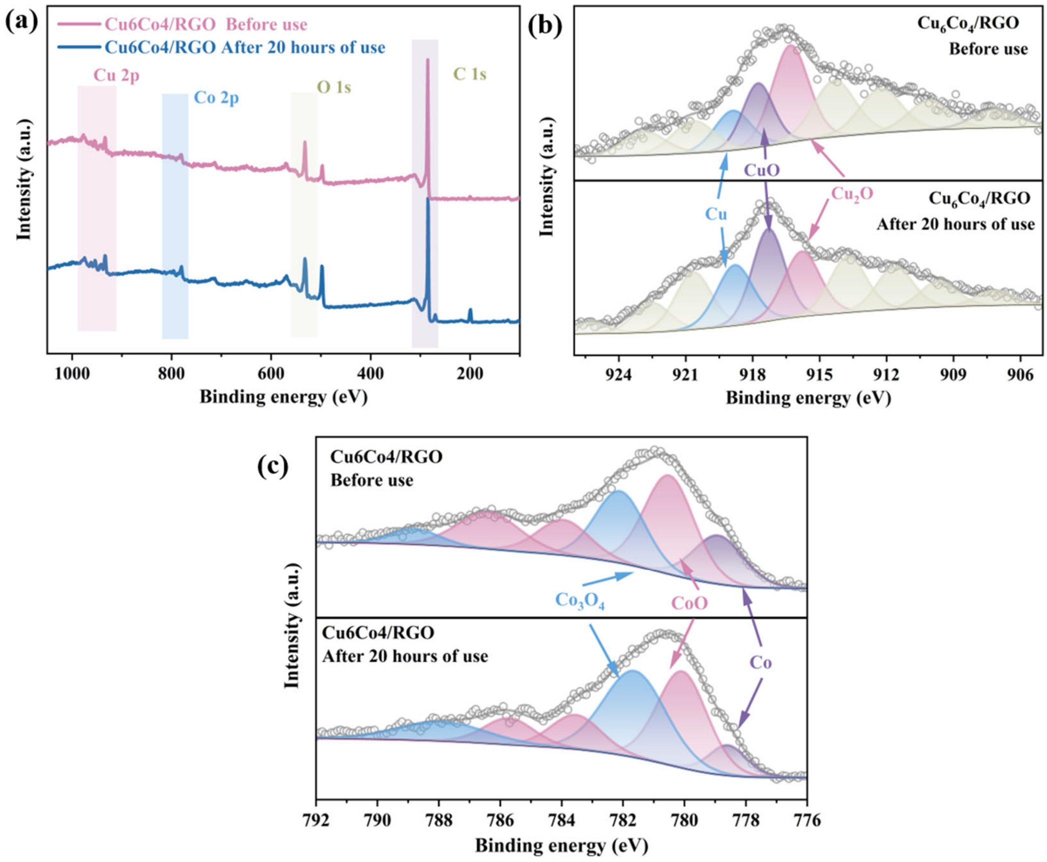

Figure S17. XPS analysis of Cu_6Co_4/RGO before and after 20 hours of electrolysis. (a) Full survey spectrum. (b) Cu 2p spectrum. (c) Cu LMM Auger spectrum. (d) Co 2p spectrum.

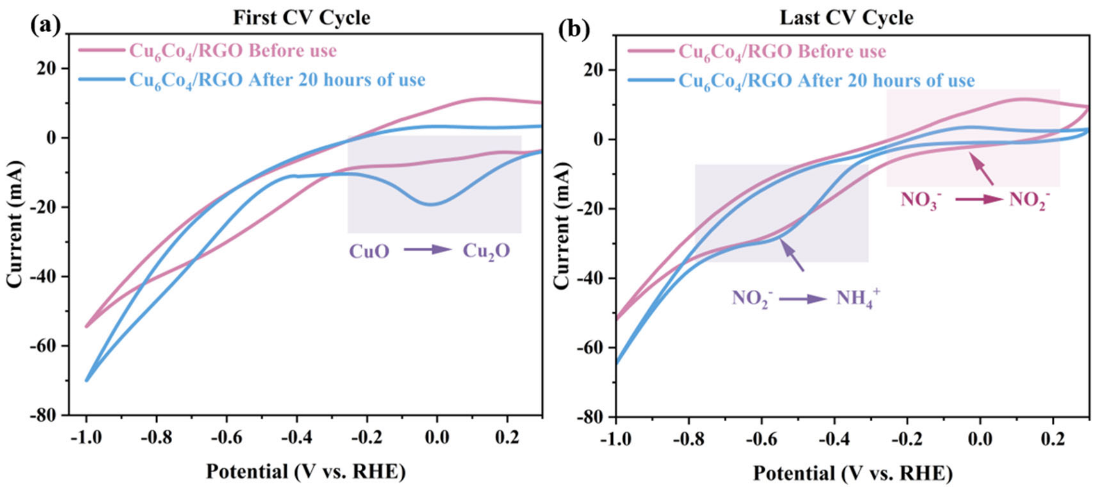

Figure S18. Electrochemical performance of Cu_6Co_4/RGO before and after electrolysis: (a) First-cycle CV results; (b) Last-cycle CV results.

Table S1. Statistical table of the proportion of carbon and oxygen on the surface of materials after hydrothermal treatment at different temperatures

Sample name	C（%）	O（%）	C:O
GO	66.97	33.03	2.03
GO-100℃	66.12	33.88	1.95
GO-120℃	65.62	34.38	1.88
GO-140℃	59.21	40.79	1.45
GO-160℃	56.12	43.88	1.27
GO-180℃	49.36	50.64	0.98

Table S2 Statistical table of peak area ratio of surface oxygen-containing functional groups after hydrothermal treatment at different temperatures

Sample name	C-O（%）	C=O（%）	O-C=O（%）
GO	80.50	19.50	0.00
GO-100℃	58.17	41.83	0.00
GO-120℃	46.42	28.08	25.50
GO-140℃	28.64	20.99	50.37
GO-160℃	24.34	19.05	56.15
GO-180℃	18.00	52.08	29.92

Table S3. ICP test results of RGO load with different Cu/Co ratios

Sample name	Cu（mg/L）	Co（mg/L）	Cu:Co
Cu/RGO	21.12	0.00	\
Cu_8Co_2/RGO	16.48	3.59	4.59
Cu_6Co_4/RGO	13.65	9.36	1.46
Cu_5Co_5/RGO	10.28	9.98	1.03
Cu_4Co_6/RGO	7.01	11.39	0.61
Cu_2Co_8/RGO	3.74	15.96	0.23
Co/RGO	0.00	14.84	\

Table S4 Detection of Cu Co element ratio of samples before and after electrolysis

Sample name	Cu（mg/L）	Co（mg/L）	Cu:Co
Cu_6Co_4/RGO Before electrolysis	13.65	9.36	1.46
Cu_6Co_4/RGO After electrolysis	12.98	9.06	1.43

Table S5 Performance comparison of Cu_6Co_4/RGO with other catalytic materials

Sample name	Potential (vs. RHE)	N-NH_4^+ selectivity	N-NH_4^+ FE
Fe_3C@NG-10[1]	-0.5 V	\	94.03%
$RGO_{2.92}$@CoAg[2]	-0.43 V	95.20%	95.10%
BCN-CuCo[3]	-0.69 V	\	70.30%
Fe-RGO[4]	-0.6 V	92.65%	89.86%
Co-TiO_2/GO[5]	-1.1V	41.2%	56.50%
NiCu@NC/NF[6]	-0.6 V	92.61 %	\
Mo_2C/ CNT-RGO[7]	-0.6 V	\	82.76%
V_2O_4/rGO[8]	-0.2 V	78%	79%
Fe/Cu-HNG[9]	-0.5V	91.10%	92.51%
Ru_{nano}-Based/rGO[10]	-0.2V	\	2.1%
This work Cu_6Co_4/RGO	-0.6V	99.86%	96.54%

References

1. Rao, T.; Zhan, J.; Du, Y.; Zhang, L.; Yu, F. Tuning Nitrogen Configurations in Nitrogen-Doped Graphene Encapsulating Fe_3 C Nanoparticles for Enhanced Nitrate Electroreduction. *ChemSusChem* **2025**, e202402460, doi:10.1002/cssc.202402460.
2. Liu, Z.; Huang, X.; Liu, X.; Liu, J.; Wang, M.; Ding, T.; Yan, L.; Zhang, Z.; Shi, G. Electrochemical Synthesis of Metasequoia-Like Reduced Graphene Oxide Coated Cobalt-Silver Catalyst for Stable and Efficient Electrocatalytic Nitrate Reduction to Ammonia. *Small* **2025**, *21*, 2408566, doi:10.1002/smll.202408566.
3. Li, Y.; Ji, S.; Fan, Y.; Duan, T.; Zhang, Y. The Charge Redistribution Induced by Cu-Co Bimetallic Synergies Efficiently Promotes Electrochemical Reduction to Ammonia. *Applied Surface Science* **2025**, *696*, 162950, doi:10.1016/j.apsusc.2025.162950.
4. Li, S.; Yan, J.; Liu, M.; Su, H. Localized Enriching Nitrate/Proton on Reconstituted Fe Nanoparticles Boosting Electrocatalytic Nitrate Reduction to Ammonia. *Journal of Energy Chemistry* **2025**, *103*, 682–691, doi:10.1016/j.jechem.2024.12.011.
5. Lin, P.; Chen, R.; Xu, S.; Xia, X.; Zhao, F.; Ren, X.; Lu, Y.; Gao, L.; Bao, J.; Liu, A. Efficient Co and GO Co-Doped TiO2 Catalysts for the Electrochemical Reduction of Nitrate to Ammonia. *CATALYSIS SCIENCE & TECHNOLOGY* **2025**, *15*, 1445–1455, doi:10.1039/d4cy01228c.
6. He, L.; Yao, F.; Zhong, Y.; Tan, C.; Hou, K.; Pi, Z.; Chen, S.; Li, X.; Yang, Q. Achieving High-Performance Electrocatalytic Reduction of Nitrate by N-Rich Carbon-Encapsulated Ni-Cu Bimetallic Nanoparticles Supported Nickel Foam Electrode. *Journal of Hazardous Materials* **2022**, *436*, 129253, doi:10.1016/j.jhazmat.2022.129253.
7. Jang, S.E.; Kim, J.Y.; Youn, D.H. Enhanced Electrochemical Nitrate Reduction to Ammonia with Nanostructured Mo_2 C on Carbon Nanotube-Reduced Graphene Oxide Hybrid Support. *Dalton Trans.* **2024**, *53*, 18141–18147, doi:10.1039/D4DT02817A.
8. Chen, S.; Liang, Y.; Bu, M.; Dai, Z.; Shi, J. V2O4 Nanowires/rGO Hybrid for Electrocatalytic Nitrogen Fixation. *Catal Lett* **2024**, *154*, 1795–1805, doi:10.1007/s10562-023-04415-x.
9. Zhang, S.; Wu, J.; Zheng, M.; Jin, X.; Shen, Z.; Li, Z.; Wang, Y.; Wang, Q.; Wang, X.; Wei, H.; et al. Fe/Cu Diatomic Catalysts for Electrochemical Nitrate Reduction to Ammonia. *Nat Commun* **2023**, *14*, 3634, doi:10.1038/s41467-023-39366-9.
10. Sun, W.; Sahin, N.E.; Sun, D.; Wu, X.; Munoz, C.; Thakare, J.; Aulich, T.; Zhang, J.; Hou, X.; Oncel, N.; et al. One-Pot Synthesis of Ruthenium-Based Nanocatalyst Using Reduced Graphene Oxide as Matrix for Electrochemical Synthesis of Ammonia. *ACS Appl. Mater. Interfaces* **2023**, *15*, 1115–1128, doi:10.1021/acsami.2c18413.
